# Stabilization of E2-EPF UCP protein is implicated in hepatitis B virus-associated hepatocellular carcinoma progression

**DOI:** 10.1007/s00018-019-03066-9

**Published:** 2019-03-22

**Authors:** Jung Hwa Lim, Dae-Ghon Kim, Dae-Yeul Yu, Hyun Mi Kang, Kyung Hee Noh, Dae-Soo Kim, Dongmin Park, Tae Kyung Chang, Dong-Soo Im, Cho-Rok Jung

**Affiliations:** 1 Gene Therapy Research Unit, Korea Research Institute of Bioscience and Biotechnology (KRIBB), Daejeon, Republic of Korea; 2 University of Science and Technology, Daejeon, Republic of Korea; 3 Research Institute of Clinical Medicine, Chonbuk National University Medical School and Hospital, Jeonju, Republic of Korea

**Keywords:** HBx, Tumor progression, pVHL, HIF, Ubiquitination

## Abstract

Hepatitis B virus (HBV) X protein (HBx) is associated with hepatocarcinogenesis. E2-EPF ubiquitin carrier protein (UCP) catalyzes ubiquitination of itself and von Hippel–Lindau protein (pVHL) for degradation and associates with tumor growth and metastasis. However, it remains unknown whether HBx modulates the enzyme activity of UCP and thereby influences hepatocarcinogenesis. Here, we show that UCP is highly expressed in liver tissues of HBx-transgenic mice, but not non-transgenic mice. UCP was more frequently expressed in HBV-positive liver cancers than in HBV-negative liver cancers. HBx binds to UCP specifically and serotype independently, and forms a ternary complex with UCP and pVHL. HBx inhibits self-ubiquitination of UCP, but enhances UCP-mediated pVHL ubiquitination, resulting in stabilization of hypoxia-inducible factor-1α and -2α. HBx and UCP stabilize each other by mutually inhibiting their ubiquitination. HBx promotes cellular proliferation and metastasis via UCP. Our findings suggest that UCP plays a key role in HBV-related hepatocarcinogenesis.

**Electronic supplementary material:**

The online version of this article (10.1007/s00018-019-03066-9) contains supplementary material, which is available to authorized users.

## Introduction

Chronic infection with hepatitis B virus (HBV) is epidemiologically linked to the development of hepatocellular carcinoma (HCC); over 80% of HCCs are related to viral hepatitis and more than half are related to HBV [8]. HBV encodes the X protein (HBx) [18]. HBx possesses numerous activities, including carcinogenic ability, via interactions with or modulations of multiple host factors involved in gene transcription, signal transduction, cell cycle, apoptosis, and DNA repair [5]. Accumulating evidences suggest that HBx is a tumorigenic factor. Nevertheless, most of HBx-transgenic (TG) mice do not develop liver tumors spontaneously [40], but only some reported that HCC occurred in 50% of the liver of HBx-TG mice at the age of 11–13 months [19, 50]. It has been unclear how this inconsistency arises [40]. Thus, the precise role of HBx in tumorigenesis needs to be elucidated to seek a novel therapeutic modality for HBV-related liver cancer patients.

The von Hippel–Lindau protein (pVHL) acts as the substrate-recognition module of the E3 ubiquitin ligase complex, which targets hypoxia-inducible factor-1α (HIF-1α) and HIF-2α for degradation [30]. HIF-1α or HIF-2α and HIF-1β form heterodimeric HIF transcription factors, which are implicated in angiogenesis and metastasis [39]. HBx stabilizes HIF-1α by interfering with the interaction between pVHL and HIF-1α and leads to angiogenesis in hepatocarcinogenesis [28], [49]. However, the molecular mechanism of HIF-1α stabilization by HBx is unclear.

E2-EPF ubiquitin carrier protein (UCP) is a member of the E2 ubiquitin conjugase family and catalyzes E3-independent and E3-dependent ligation of ubiquitin [24]. We have shown previously that UCP stabilizes HIF-1α by targeting pVHL for degradation and is associated with cell proliferation, angiogenesis, and metastasis [15, 22, 23]. UCP (UBE2S) confers E2 enzyme activity to the anaphase-promoting complex (APC), an E3 ubiquitin ligase [46]. As UCP stabilized HIF-α proteins by decreasing the pVHL level [15], we tested whether HBx-mediated catalytic regulation of the UCP activity is associated with the stability of HIF-α proteins and thereby contributes to the promotion of hepatocellular carcinoma. Here, we show that HBx stabilizes UCP by inhibiting its self-ubiquitination and increases the levels of HIF-α proteins by enhancing UCP-mediated pVHL ubiquitination. Therefore, UCP is actively involved in HBx-mediated tumor growth and metastasis.

## Results

### UCP is frequently detected in HBV-positive human HCC

**Fig. 1:**
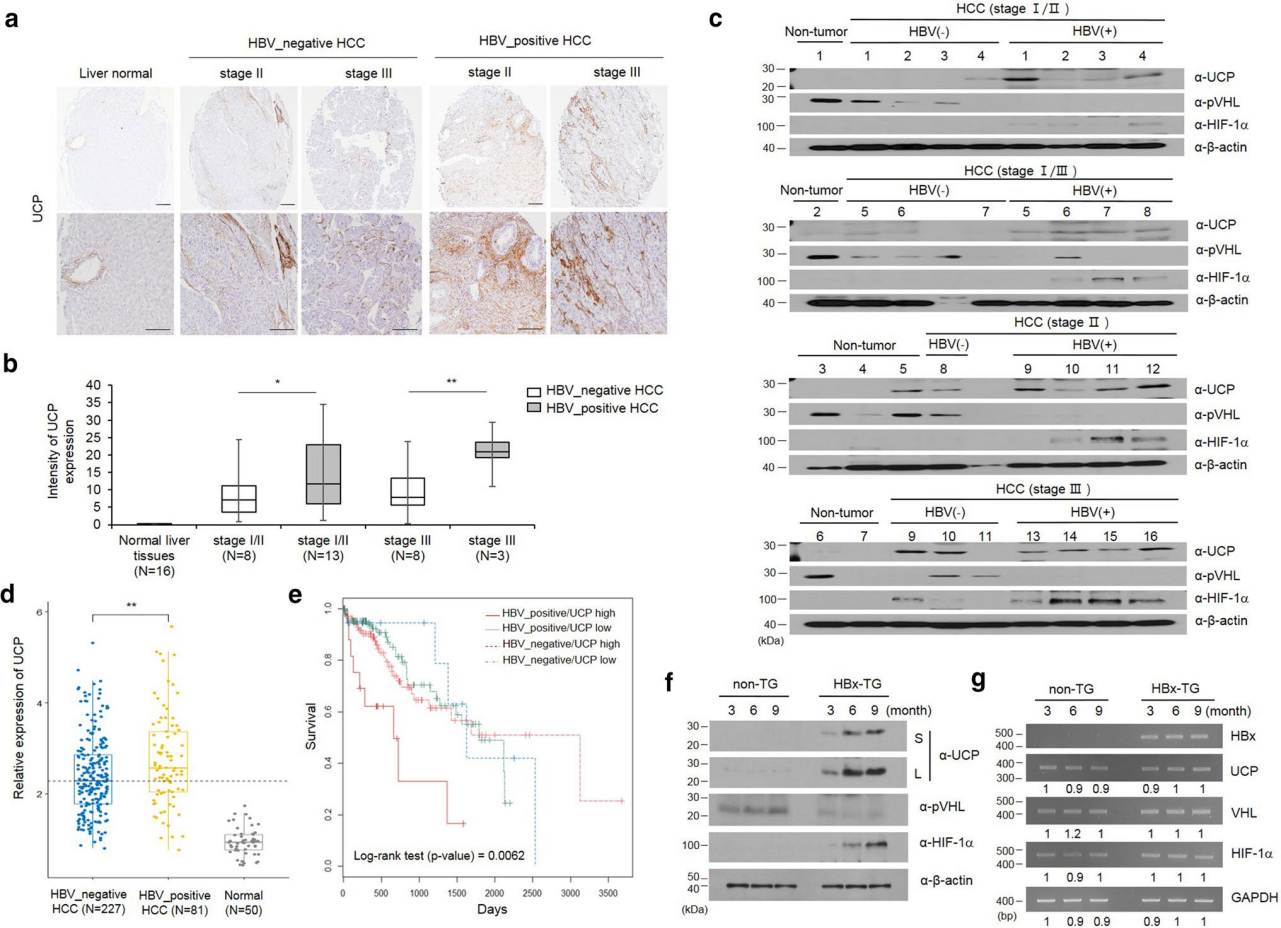
UCP is highly expressed in the liver tissues of HBx-transgenic mice and is frequently detected in HBV-positive HCCs. **a** The representative image shows that UCP was strongly expressed in late stage of HBV-positive HCCs compared to HBV-negative HCCs. **b** UCP staining intensity was quantified using Image J software. **c** We immunoblotted protein extracts from HBV-positive (+) or HBV-negative (−) HCC tissues of the indicated stages and from non-tumor tissues adjacent to or apart from HCC tissues as indicated. **d** The expression of UCP was assessed with liver hepatocellular carcinoma (TCGA-LIHC) RNA-seqv2 data (HTSeq-FPKM) and clinical datasheets were obtained from the TCGA data portal. **e** Overall survival rate was assessed in hepatocellular carcinoma (HCC) of HBV-positive or HBV-negative patients. We prepared whole cell lysates or total RNAs from liver tissues of TG or non-TG mice at the indicated age and analyzed them by WB (**f**) or RT-PCR (**g**) as indicated. RT-PCR products were quantified by densitometry and the results are expressed relative to the band intensity of the first lane in **b**, which was arbitrarily defined as 1. *S* short exposure, *L* long exposure. We repeated the experiments of this figure twice; representative data are shown (i.e., *p < 0.05, **p < 0.01)

To determine whether there is a correlation between HBV-positive HCC and UCP expression, we examined UCP levels in human non-tumor liver tissues as well as HBV-positive and -negative HCCs (Fig. 1a–c). IHC assay was performed in microarray containing normal liver and HCC tissues (16 pairs of normal liver tissues, 16 pairs of HBV-positive and 16 pairs of HBV-negative HCC tissues). As shown, the expression of UCP was not detectable in normal liver tissues, whereas UCP expression in late stage of HBV-positive HCC tissues was strongly increased compared to HBV-negative HCC tissues (Fig. 1a, b). The expression of UCP protein was analyzed by WB in HBV-negative or -positive HCC and normal liver tissues (7 of normal liver tissues, 16 of HBV-positive and 11 of HBV-negative HCC tissues). UCP was detected in 16 of 16 HBV-positive HCCs (100%), but it was only detected in 6 of 11 HBV-negative HCCs (54%). UCP and HIF-1α proteins were identified in one and zero out of seven non-tumor samples, respectively, while pVHL was detected in six (85%) of seven non-tumor samples. HIF-1α was detected in 13 (81%) of 16 HBV-positive HCCs and in 2 (18%) of 11 HBV-negative HCCs. The presence of pVHL in 8 (72%) of 11 HBV-negative HCCs may account for the low rate of HIF-1α detection in the HBV-negative samples. These data suggest that HBV-positive HCC is associated with increased protein levels of UCP and HIF-1α (Fig. 1c).

In a previous study, the survival rate and the development of hepatocellular carcinoma were monitored for 30 months in patient with cirrhosis associated with HBV infection. The rate of HBV-mediated HCC development was 1.5-fold higher in patients with HBV infection (65%, *N* = 19/25) than without HBV (46%, *N* = 16/35) and the 5-year survival rates were 9% in HBV-positive cirrhosis group and 34% in HBV-negative cirrhosis group, respectively [10]. To confirm whether the difference of UCP expression was due to HBV infection in HCC patients, we assessed whether UCP expression was significantly increased in HBV-positive HCC patients compared to HBV-negative HCC patients with liver hepatocellular carcinoma (TCGA-LIHC) RNA-seqv2 data (Fig. 1d, *p* > 0.01). Further, to determine whether there was a correlation between UCP expression and survival rate, we assessed the survival rate using clinical datasheet from the TCGA data portal. Our results showed that HBV-positive patients with increased expression of UCP showed significantly lower survival rates compared to the other groups (Fig. 1e, *p* > 0.01). These data suggest that increased expression of UCP, as it relates to HBV infection, activates the malignant transformation of HCC progression and results in low survival rate.

### UCP is highly expressed in HBx-transgenic mouse

To determine whether HBx influences UCP, we examined protein and mRNA levels of UCP in liver tissues from HBx-TG and non-TG mice (Fig. 1f, g). UCP was clearly detected in the liver tissues of HBx-TG mice along with the age, whereas it was barely detected in the liver tissues of non-TG mice (Fig. 1f). pVHL protein levels were much lower in HBx-TG mouse livers compared to non-TG livers. HIF-1α was detected in liver tissues where UCP was evidently detected. There were no significant changes in the mRNA levels of these proteins in the liver tissues of HBx-TG and non-TG mice (Fig. 1g). These results suggest that HBx is associated with increased UCP expression at the protein level in the mouse liver, in which UCP likely regulates the protein levels of pVHL and HIF-1α.

### HBx decreases pVHL level by increasing UCP level in cells and regulates HIF-α levels via pVHL

**Fig. 2:**
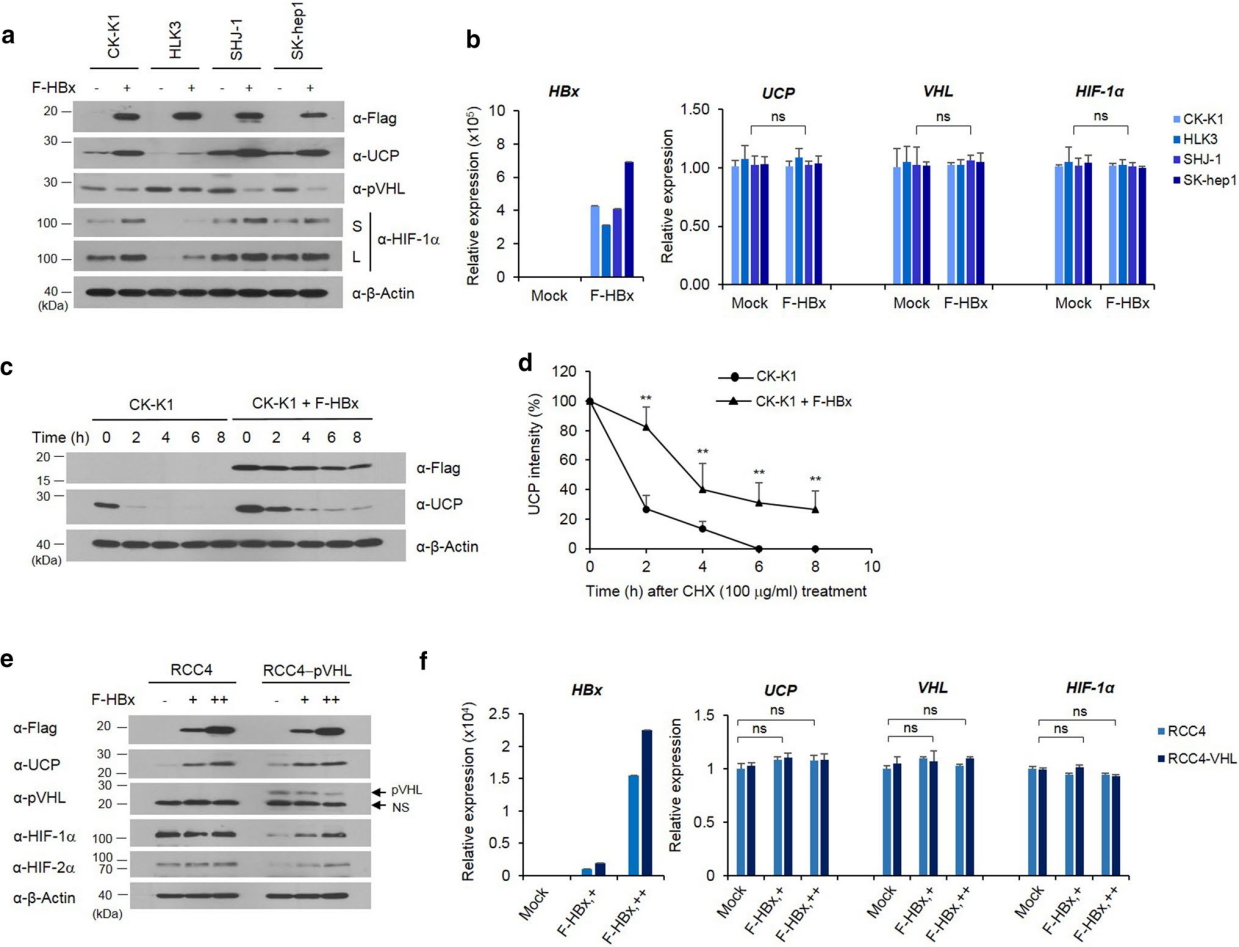
HBx decreases pVHL level by increasing UCP level in cells and regulates HIF-α levels via pVHL. We transfected the indicated cells with FLAG-tagged HBx (F-HBx) expression vector (0, 5 μg, −, +), incubated them for 24 h, and analyzed them by WB (**a**) or qRT-PCR (**b**) as indicated. Representative results of at least three independent experiments are shown. *S* short exposure, *L* long exposure. **c**, **d** We transfected or not CK-K1 cholangiocarcinoma cells with 5 μg of F-HBx expression vector, incubated them for 24 h, treated them with cycloheximide (CHX), harvested them at the indicated times, and analyzed them by WB as indicated. We repeated this experiment three times; representative data are shown in **c**. UCP bands were quantified by densitometry and are expressed relative to the band intensity of ‘time 0’, which was arbitrarily defined as 100 (**d**). Data of **d** are mean ± SD. We transfected the indicated cells with F-HBx expression vector (0, 5, 15 μg, −, +, ++), prepared whole cell lysates or total RNAs, and analyzed them by WB (**e**) or qRT-PCR (**f**) as indicated. Protein and mRNA levels were quantified by densitometry. Protein levels are expressed relative to the band intensity of the first or fourth (pVHL panel) lane in **e**, which was arbitrarily defined as 1. Representative results of at least two independent experiments are shown

Having determined that HBV infection, particularly HBx, is associated with the increased expression of UCP in vivo (Fig. 1), we examined whether HBx increases UCP expression in cells. HBx expression increased the protein levels of UCP and HIF-1α in various HCC cell lines and decreased the pVHL level (Fig. 2a). HBx expression did not affect the mRNA levels of these molecules (Fig. 2b and Supplementary Fig. 2a). HBx expression prolonged half-life of UCP protein from ~ 1.3 to ~ 3.5 h (Fig. 2c, d).

Renal carcinoma cell line (RCC) 4 lacks wild-type pVHL and expresses both HIF-1α and HIF-2α [26]. Using RCC4 cell lines with or without reintroduced pVHL [26], we examined whether HBx-mediated increases of HIF-α proteins depend on pVHL levels. HBx expression increased UCP levels in both RCC4 and RCC4-pVHL cells in a dose-dependent manner, and decreased pVHL levels in RCC4-pVHL cells (Fig. 2e). HBx increased HIF-1α and HIF-2α expression in RCC4-pVHL cells, but it did not greatly affect the levels of these proteins in RCC4 cells (Fig. 2e). The mRNA levels of these proteins were not greatly affected by HBx expression (Fig. 2f and Supplementary Fig. 2b). Because UCP binds to and targets pVHL for degradation [15], these results suggest that HBx-mediated increases of UCP determines the stability of HIF-α proteins via pVHL. Similar findings were obtained in 786-0 cells with or without pVHL expression (Supplementary Fig 1).

### HBx interacts with UCP and forms a ternary complex with UCP and pVHL

**Fig. 3:**
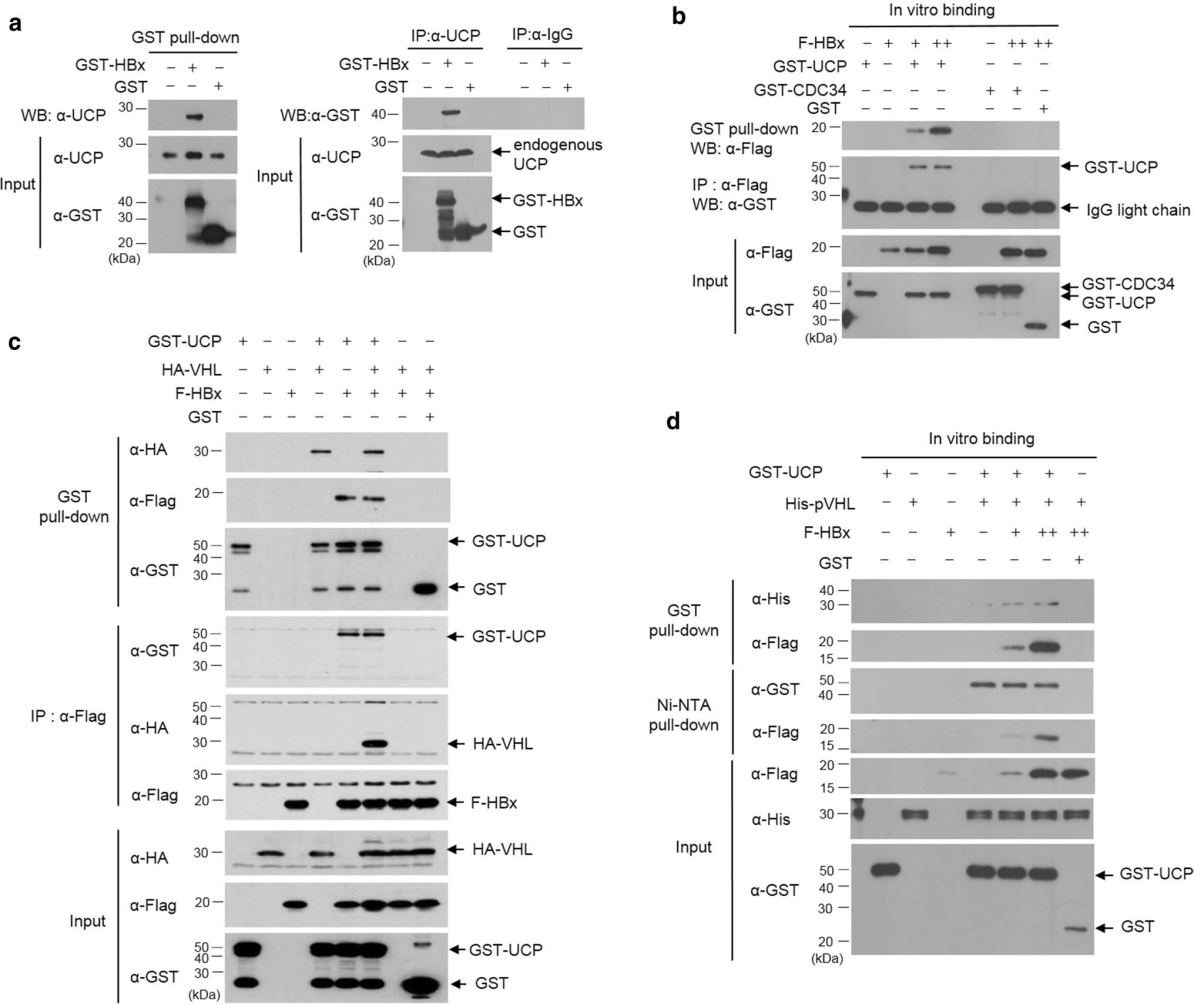
HBx interacts with UCP and forms a ternary complex with UCP and pVHL. **a** We transfected or not HLK3 cells with 5 μg of GST (glutathione *S*-transferase)-HBx or GST expression vector, incubated them for 24 h, and performed GST pull-down or immunoprecipitation (IP) assays as indicated. **b** We mixed GST-UCP (1 μg), F-HBx (0, 0.5, 2 μg, −, +, ++), GST-CDC34 (1 μg), and/or GST (1 μg) proteins, and performed GST pull-down or IP assays as indicated. **c** We transfected 293T cells with GST-UCP, HA-VHL, F-HBx, and/or GST expression vector (10 μg each), incubated them for 24 h, and performed GST pull-down and IP assays as indicated. **d** We mixed GST-UCP (1 μg), His-pVHL (1 μg), F-HBx (0.5, 2 μg, +, ++), and/or GST (1 μg) proteins, incubated them at 4 °C for 1 h, and performed pull-down assays as indicated. We repeated the experiments of **a**–**d** twice; representative data are shown

To elucidate how HBx increases UCP levels, we examined whether HBx binds to UCP. HBx interacted with endogenous UCP in cells and vice versa (Fig. 3a). In vitro, HBx interacted with UCP but not with cell division cycle 34 (CDC34) (Fig. 3b), which belongs to the E2 ubiquitin conjugase family [43]. HBx did not interact with pVHL in vitro and in cells (Fig. 3c, d). Nevertheless, HBx formed a ternary complex with UCP and pVHL, presumably because UCP interacted with pVHL (Fig. 3c, d, and Supplementary Fig. 3d, e). To identify the binding site of HBx in UCP and vice versa, we constructed various deletion mutants of HBx and UCP and performed GST pull-down assay with them. The N-terminus of HBx (16–50 amino acids [aa]), but not the transcription factor binding domain (51–154 aa), was required for the interaction of HBx with UCP. Full-length UCP was involved in the interaction with HBx or pVHL (Supplementary Fig. 3b, c and e).

The amino-terminus regulatory region (1–50 aa) of HBx to which UCP binds has been suggested to regulate the stability of HBx protein [31]. Indeed, the peptidyl prolyl isomerase (Pin 1) binds to HBx through the ser41-pro motif in its amino-terminal region and stabilizes HBx [32]. The ser41-pro motif is present in HBx serotype ayw, but not HBx adr (Supplementary Fig. 3a). The N-termini (1–50 aa) of HBx from both serotypes adr and ayw were found to equally bind to UCP (Supplementary Fig. 3f). These results suggest that HBx serotype-independently interacts with UCP and the amino-terminus regulatory domain (1–50 aa) of HBx is essential for the interaction.

### HBx and UCP stabilize each other by mutually inhibiting their ubiquitination

**Fig. 4:**
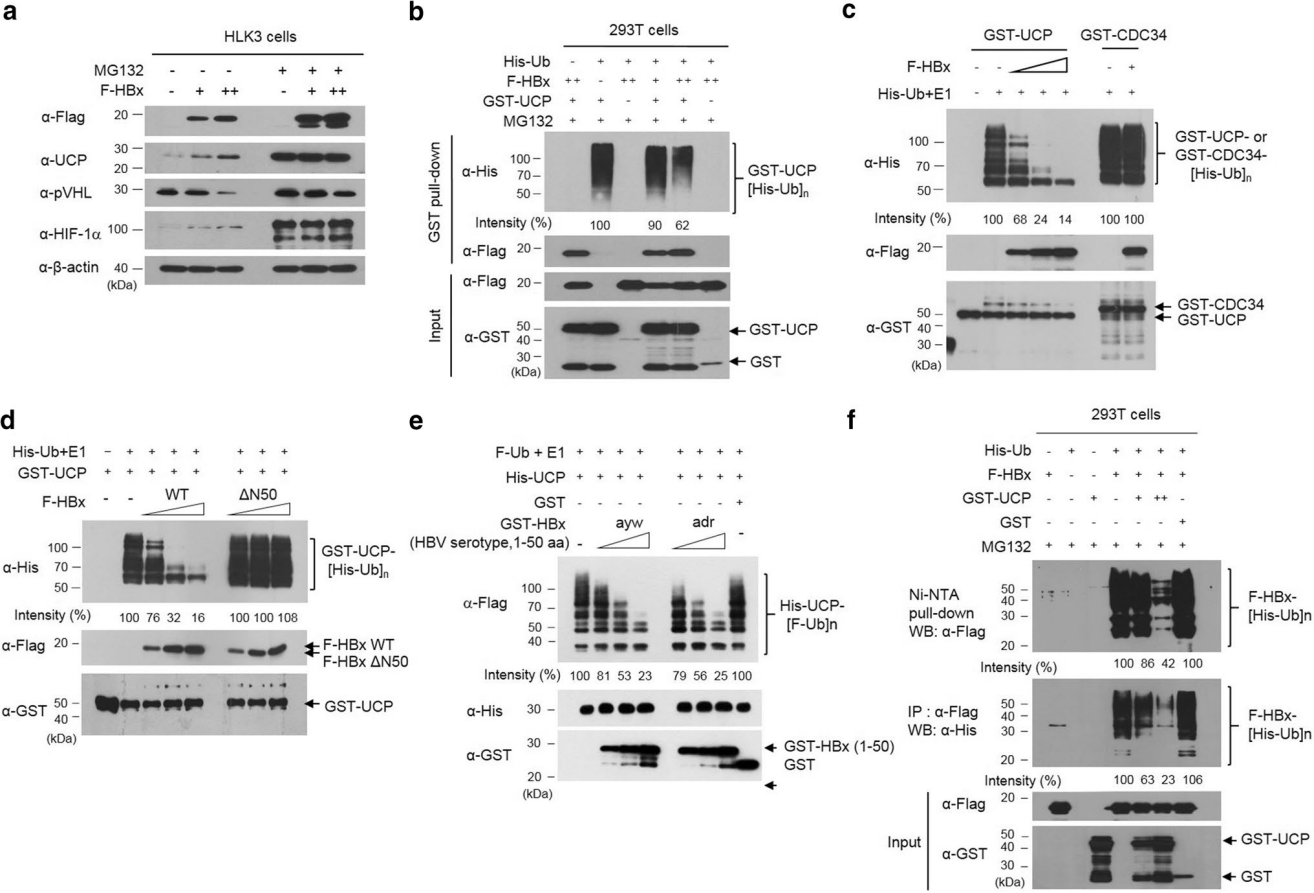
HBx and UCP stabilize each other by mutually inhibiting their ubiquitination. **a** We transfected or not HLK3 cells with F-HBx expression vector (0, 5, 15 μg, −, +, ++), incubated them for 32 h, further incubated them in the presence or absence of 10 μM MG132 for 16 h, and immunoblotted them as indicated. **b** We transfected 293T cells with F-HBx (0, 5, 15 μg, −, +, ++), GST-UCP (5 μg), His-ubiquitin (Ub) (5 μg), or GST (5 μg) expression vector, incubated them for 32 h, treated them with or without 10 μM MG132 for 16 h, and performed GST pull-down assays as indicated. GST-UCP bands conjugated with His-Ub were quantified by densitometry and are expressed relative to the band intensity of ubiquitinated GST-UCP in lane 2, which was defined arbitrarily as 100. **c** We performed an in vitro ubiquitination assay with GST-UCP, GST-CDC34 (0.3 μg), and F-HBx (0.5, 1, 2 μg, +, ++, +++) proteins, and immunoblotted the reaction mixtures as indicated. GST-UCP bands conjugated with His-Ub were quantified by densitometry and are expressed relative to the band intensity of ubiquitinated GST-UCP in lane 2, which was defined arbitrarily as 100. **d** We performed an in vitro ubiquitination assay with GST-UCP, F-HBx WT (0, 0.5, 1, 2 μg), and F-HBxΔN50 (0.5, 1, 2 μg) proteins, and immunoblotted the assay mixtures as indicated. GST-UCP bands conjugated with His-Ub were quantified by densitometry and are expressed relative to the band intensity of ubiquitinated GST-UCP in the absence of F-HBx (lane 2), which was defined arbitrarily as 100. **e** We performed an in vitro ubiquitination assay with His-UCP, GST-HBx (1–50 aa) ayw or adr (0.5, 1, 2 μg), or GST (2 μg) proteins, and analyzed the reaction mixtures as indicated. **f** UCP inhibits ubiquitination of HBx in cells. We transfected 293T cells with His-Ub (5 μg), F-HBx (5 μg), GST-UCP (0, 5, 15 μg, −, +. ++), or GST (15 μg) expression vector, incubated them for 32 h, treated them with 10 μM MG132 for 16 h, and prepared cell lysates. We performed Ni–NTA pull-down and IP assays with cell lysates as indicated. We repeated the experiments of **a**–**d**, **f** three times and that of **e** twice; representative data are shown

To examine how HBx stabilizes UCP, UCP expression was examined in HLK3 cells with and without HBx expression and in the presence or absence of the proteasome inhibitor MG132 (Fig. 4a). UCP was stabilized in the presence of MG132, regardless of HBx expression, suggesting that the UCP level is regulated by ubiquitin-mediated proteolysis in cells. UCP was ubiquitinated in cells, and HBx expression substantially decreased the level of ubiquitinated UCP (Fig. 4b). UCP self-ubiquitinates [24] and this ability may be a mechanism by which UCP levels are regulated in cells. HBx protein inhibited self-ubiquitination of UCP in vitro but not CDC34 (Fig. 4c), which also has self-ubiquitination activity in vitro and in vivo [43]. HBx also inhibited UCP-mediated ubiquitination of a catalytically inactive UCP (C95A) mutant (Supplementary Fig. 4), in which cysteine-95 is mutated to alanine [15]. The N-terminus domain (1–50 aa) of HBx ayw or adr, but not HBxΔN50 (51–154 aa), inhibited self-ubiquitination of UCP (Fig. 4d, e). These results suggest that HBx, particularly its N-terminus regulatory domain (1–50 aa), stabilizes UCP by inhibiting UCP self-ubiquitination.

HBx turnover has been shown to be ubiquitin dependent [12]. Indeed, the protein level of F-HBx increased in the presence of MG132 (Fig. 4a). We observed that UCP expression in the NIH3T3 cell line expressing HBx greatly increased the protein level of F-HBx (Supplementary Fig. 6d). UCP expression inhibited the ubiquitination of HBx in cells (Fig. 4f) and prolonged the half-life of HBx from ~ 1.6 to ~ 4.5 h (Supplementary Fig. 5). We observed that UCP expression in the NIH3T3 cell line expressing HBx greatly increased the protein level of F-HBx (Supplementary Fig. 6d). These results suggest that HBx and UCP stabilize each other by mutually inhibiting their ubiquitination.

### HBx enhances UCP-mediated ubiquitination of pVHL

**Fig. 5:**
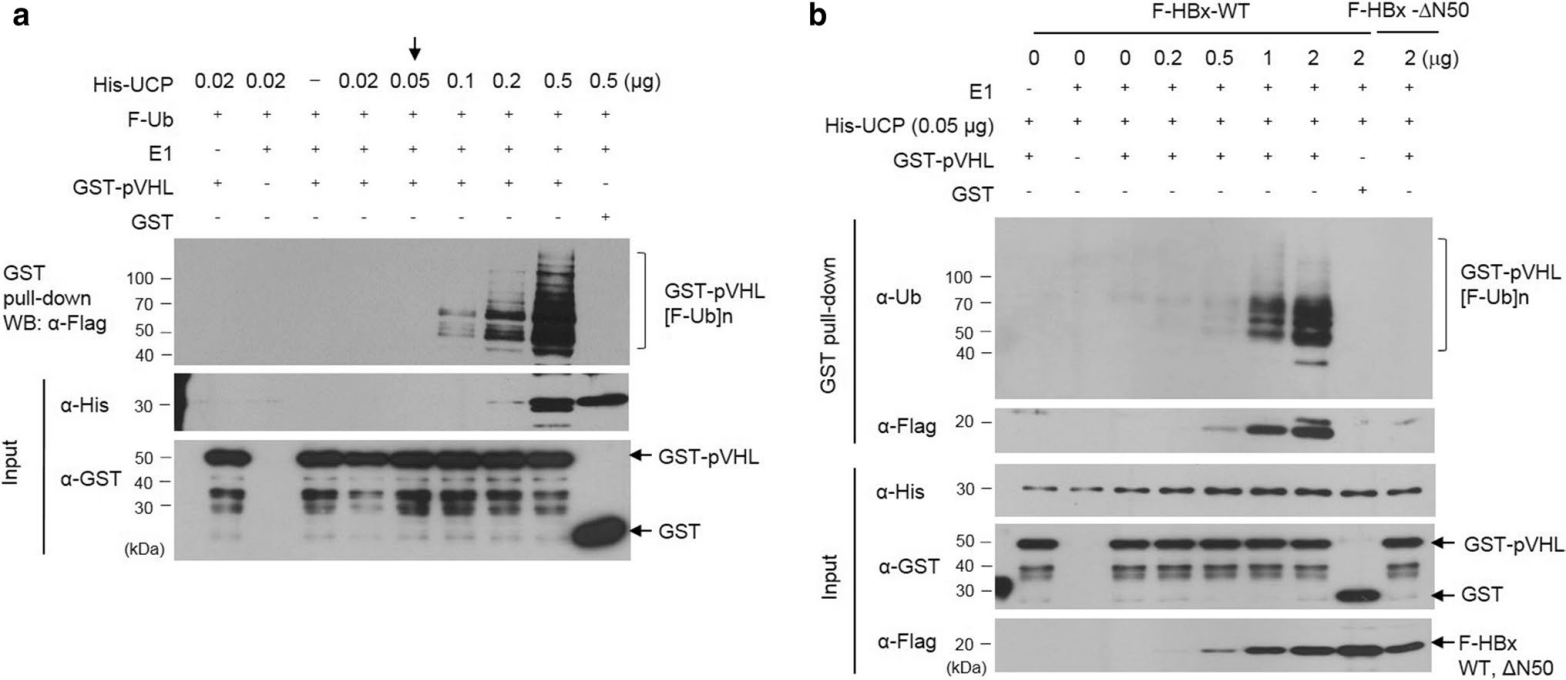
HBx enhances UCP-mediated ubiquitination of pVHL. **a** We performed an in vitro pVHL ubiquitination assay with GST-pVHL (5 μg) or GST (5 μg) at various concentrations of His-UCP, and analyzed the reaction mixtures as indicated. **b** We performed an in vitro pVHL ubiquitination assay with His-UCP (0.05 μg), GST-pVHL (5 μg), or GST (5 μg) at the indicated concentration of F-HBx WT or ΔN50, and analyzed the reaction mixtures as indicated. We repeated the experiments of **a**, **b** twice; representative data are shown

To determine whether HBx regulates the conjugase and ligase activity of UCP, we performed an in vitro pVHL ubiquitination assay with various concentrations of UCP (Fig. 5a). UCP increased pVHL ubiquitination in a concentration-dependent manner. pVHL ubiquitination was barely detected using 0.05 μg/reaction volume (50 µl) of His-UCP (Fig. 5a, indicated by an arrow). Under this condition, HBx but not HBxΔN50 stimulated the UCP-mediated ubiquitination of pVHL in a concentration-dependent manner (Fig. 5b). Thus, these results suggest that HBx can decrease pVHL level in cells by modulating the catalytic activity of UCP.

### HBx promotes cell proliferation via UCP

**Fig. 6:**
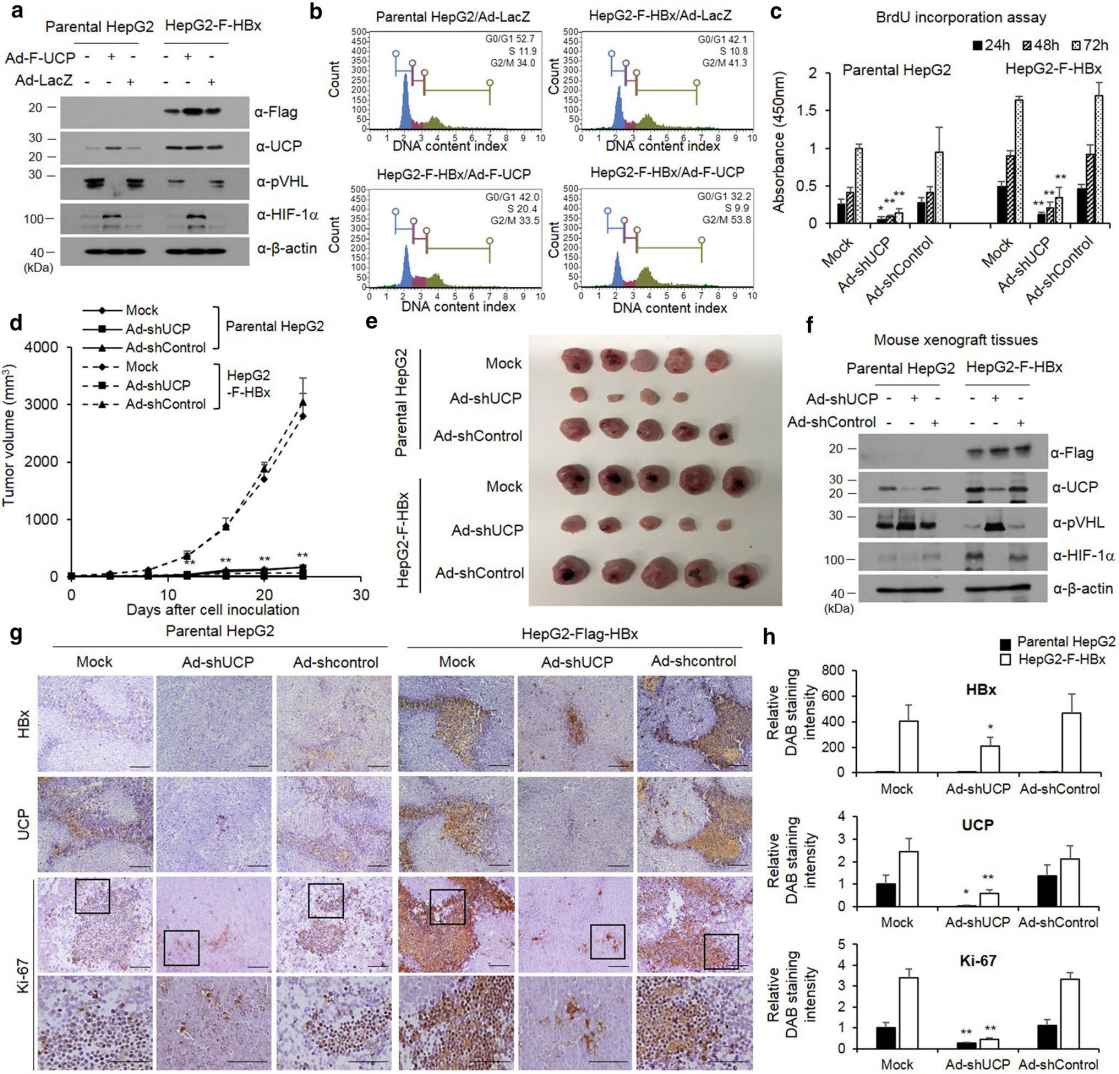
HBx promotes cell proliferation via UCP. We transduced or not parental or F-HBx-expressing HepG2 cells with Ad-F-UCP or Ad-LacZ at a MOI of 50, incubated them for 24 h, and analyzed them by WB as indicated (**a**). Cell cycle determined using Muse Cell Analyzer in the parental or F-HBx-expressing HepG2 cells with Ad-F-UCP or Ad-LacZ at an MOI of 50 (**b**). **c** We transduced or not parental or F-HBx-expressing HepG2 cells with Ad-shUCP or Ad-shControl at an MOI of 200, incubated them for 24 h, treated with BrdU, and cell viability was then determined for 3 days. We repeated this assay twice, each in triplicate. Data are mean ± SD. **d**–**f** We transduced or not parental or F-HBx-expressing HepG2 cells with Ad-shUCP or Ad-shControl at an MOI of 200, incubated them for 24 h, and injected them (2 × 10^7^ cells/mouse) into nude mice (*N* = 5 per group) subcutaneously. After cell inoculation, tumor growth was monitored by measuring tumor volume with digital caliper at the indicated times (**d**). Mice were killed at day 24, and tumors were then excised from mice and photographed (**e**). HBx–UCP–VHL–HIF axis was analyzed by western blotting in tumor xenografts (**f**). We repeated the assay of **d**–**f** twice; representative data are shown. Immunohistochemistry was performed with HBx, UCP, and Ki-67 antibodies on paraffin-embedded tumor xenografts (**g**). The relative intensity of DAB staining was quantified using Image J software, and graphed (**h**). (i.e., *p < 0.05, **p < 0.01)

To test the effect of HBx-mediated increases of UCP levels on cell proliferation, we transduced parental or HBx-expressing HepG2 cell lines with an adenoviral vector encoding F-UCP (Ad-F-UCP) and UCP–VHL–HIF signal pathway by HBx expression and assessed the results via WB (Fig. 6a). Under the same conditions, the effect of HBx and UCP on cell cycle was investigated using Muse Cell Analyzer in parental or HBx-expressing HepG2 cell lines after Ad-F-UCP or Ad-LacZ transduction (Fig. 6b). These results showed that UCP overexpression under stable expression of HBx synergistically increased cell cycle compared to HBx or UCP expressing alone.

Next, we examined the effect of HBx and UCP on cellular proliferation. HBx-expressing HepG2 cells in culture grew slower when the endogenous UCP level was decreased by Ad-shRNA (Fig. 6c). We also examined the effect of HBx-mediated UCP level increases on tumor growth in mice. HBx-expressing HepG2 cells with or without Ad-shControl rapidly increased tumor growth compared to parental HepG2 cells, and HBx-expressing HepG2 cells with Ad-shUCP strongly suppressed tumor formation (Fig. 6d, e). At 24 days after HepG2 cell inoculation, tumor xenografts were separated from mouse flanks and the HBx–UCP–VHL–HIF signal pathways were analyzed by WB (Fig. 6f). To confirm the effect of HBx-mediated increases of UCP levels on tumor growth, xenograft tumors were stained with Ki-67 in the presence or absence of HBx and UCP. As a result, Ki-67 showed increased expression in HBx-expressing HepG2 cells compared to parental HepG2 cells and was significantly reduced in HBx-expressing HepG2 treated with Ad-shUCP (Fig. 6g, h).

Additionally, we independently established NIH3T3 cell lines that stably express HBx and examined whether there was a correlation between the proliferation rate and the expression of HBx and UCP (Supplementary Fig. 6a–i). In this normal fibroblast model, HBx and UCP also promoted tumor growth synergistically. Collectively, these results suggest that UCP is actively involved in the HBx-mediated stimulation of cell proliferation.

### HBx promotes cell invasion and metastasis via UCP

**Fig. 7:**
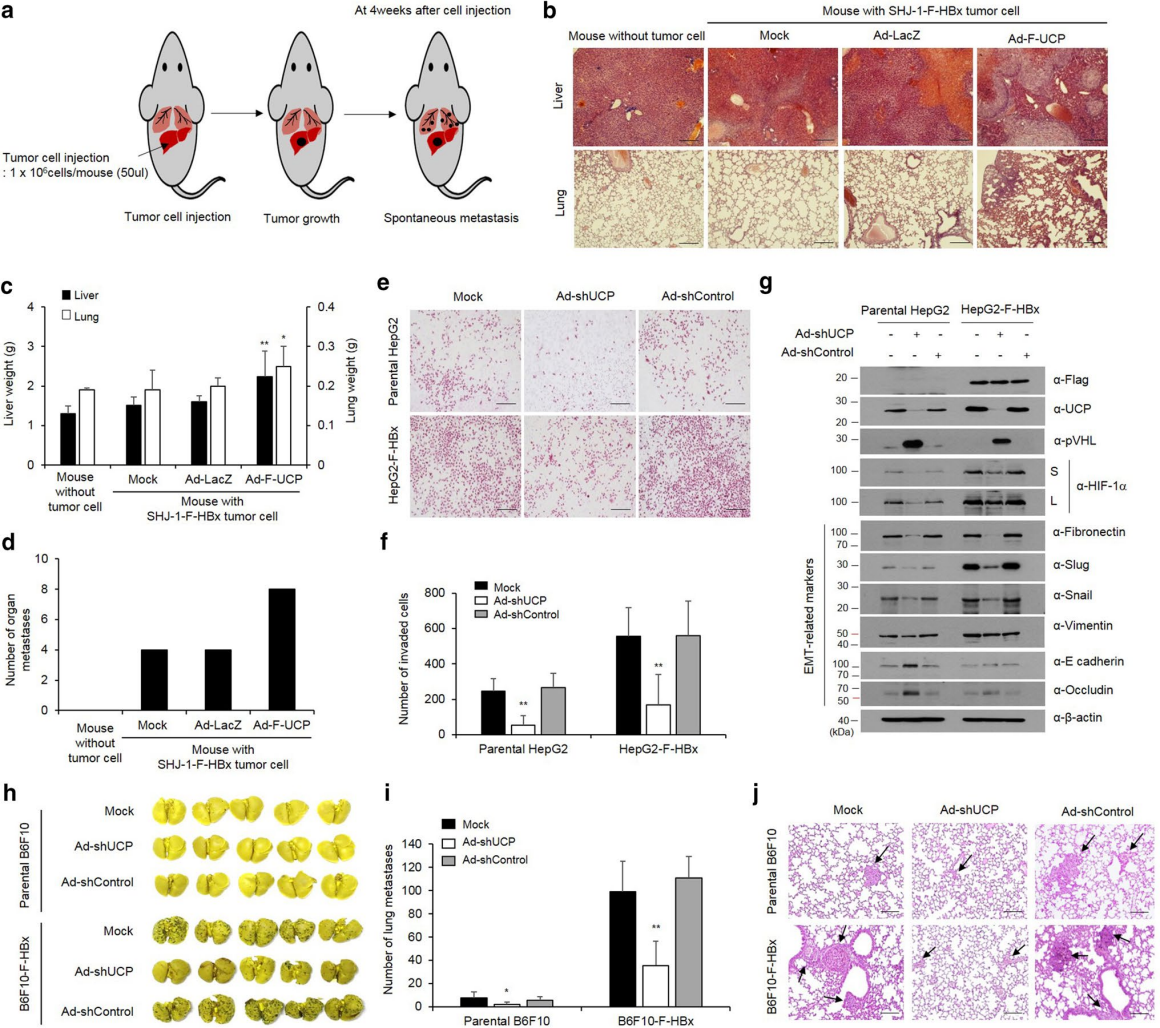
HBx promotes cell invasion and metastasis via UCP. **a** Schematic representation of experimental of orthotopic metastasis. At day 0, F-HBx-expressing SHJ-1 cells were injected into the liver of BAL b/c nude mice. **b**–**d** We transduced or not F-HBx-expressing SHJ-1 cells with Ad-F-UCP or Ad-LacZ at an MOI of 25, incubated them for 24 h, mixed them with Matrigel, and injected the mixtures into nude mouse liver (*N* = 4 mice per experimental group, *N* = 3 mice per control group). After 4 weeks, the liver and lung were excised from mice with or without cell injection, stained with H&E (scale bar 100 μm) (**b**), and weighed (**c**). The number of tumors that metastasized from the liver to the surface of lung, intestine, skin, spleen, and kidney were counted, and graphed (**d**). We repeated the experiments of **a**–**d** twice; representative data are shown. Data in Fig. 8a are mean ± SD. We transduced or not parental or F-HBx-expressing HepG2 cells with Ad-shUCP or Ad-shControl at an MOI of 200, incubated them for 48 h, and performed an invasion assay with each group of the cells in triplicate. Invaded cells were stained with H&E, and photographed under a microscope (scale bar 100 μm) (**e**). Representative images are shown. Invaded cells in **e** are counted in five random fields and graphed (**f**). Data are mean ± SD. **g** We transduced parental or F-HBx-expressing HepG2 cells with Ad-shUCP or Ad-shControl at an MOI of 200, and expression of EMT-related genes was analyzed by WB. **h**–**j** We transduced or not parental or F-HBx-expressing B6F10 cells with Ad-shUCP or Ad-shControl at an MOI of 200, incubated them for 24 h, and injected them (2 × 10^6^ cells/mouse) into nude mice (*N* = 5 per group) via the tail vein. After 2 weeks, lungs excised from mice were stained with Bouin’s solution, photographed (**h**), and embedded in paraffin. The number of tumors that metastasized to the lung was counted and graphed (**i**). We repeated the experiment of **f**, **g** twice; representative data are shown. Data in **g** are mean ± SD. Paraffin-embedded lungs were sectioned and stained with H&E (scale bar 100 μm) (**j**). Arrows indicate tumors that metastasized to the lung

To examine the effect of HBx-mediated stabilization of UCP in an orthotopic metastasis model, we grafted liver tumor cells in the liver of mice (Fig. 7a, and Supplementary Fig. 7a, b) and investigated the incidence of metastasis to other organs, including the lung. The liver and lungs excised from mice of the experimental group with additional UCP expression weighed significantly more than those obtained from the mice in the control groups, and these results were corroborated by an H&E staining (Fig. 7b, c, and Supplementary Fig. 7c, d). Metastasis was twofold more frequent in the UCP expression group than the control groups (Fig. 7d, and Supplementary Fig. 7e).

Invasion ability of HBx-expressing HepG2 cells was approximately threefold greater than that of parental cells, and Ad-shUCP abrogated the increased invasiveness of the HBx-expressing cells (Fig. 7e, f). To confirm the invasive properties of the HBx–UCP axis, we conducted an invasion assay in NIH3T3 with normal character and HBx-expressing NIH3T3 cell lines (Supplementary Fig. 8). These results showed that HBx activates the invasiveness of cancer cells through the UCP–VHL–HIF pathway. To verify whether UCP expression regulates the expression of epithelial–mesenchymal transition (EMT)-related genes in HBx-expressing cell line, we transduced parental or HBx-expressing HepG2 cell lines with an Ad-shUCP or Ad-shControl and analyzed the results by WB. These results suggest that HBx expression regulates EMT-related genes through the UCP–VHL–HIF signaling pathway (Fig. 7g). To further test whether HBx-mediated increases of UCP levels influence metastasis in mice, we performed an experimental metastasis assay using an HBx-expressing B6F10 cell line (B6F10-F-HBx). HBx expression drastically increased metastasis to the lung, and Ad-shUCP significantly reduced the increased metastasis by the HBx expression (Fig. 7h–j). These results suggest that HBx promotes cell invasion and metastasis through UCP.

## Discussions

Here, we demonstrated that HBx stabilizes UCP by inhibiting its ubiquitin-mediated proteolysis, enhances UCP-mediated pVHL ubiquitination, and thereby stabilizes HIF-1α and HIF-2α in cells, both of which are frequently overexpressed in HCC [29]. Furthermore, UCP stabilizes HBx by inhibiting its ubiquitination and is actively involved in HBx-mediated tumor growth and metastasis in cells and mice. Although viral proteins are known to interact with the ubiquitin–proteasome components [27, 37], this is the first evidence that HBx specifically modulates the catalytic activity of the E2/E3 enzyme UCP and thereby promotes hepatocarcinogenesis.

Purified E2 enzymes have been shown to self-associate into dimers or higher-order complexes in vitro [36, 43]. UCP likely recognizes itself as a substrate via homodimerization to enable self-ubiquitination. It is possible that the binding of HBx to UCP interferes with homodimerization of UCP and thereby inhibits its self-ubiquitination. HBx does not bind pVHL, but forms a ternary complex with UCP and pVHL (Fig. 3c, d) and boosts UCP-mediated pVHL ubiquitination (Fig. 5b). Thus, the inhibition of UCP self-ubiquitination by HBx and HBx/UCP-mediated enhancement of pVHL ubiquitination is a potential mechanism by which HBx increases the UCP level, effectively decreases pVHL level, and stabilizes HIF-α proteins in cells. Furthermore, this effect of HBx/UCP on stabilization of HIF-α proteins may be additionally strengthened, because HBx and UCP mutually prolong their half-life (Fig. 2 and Supplementary Fig. 5). We have shown previously that Enigma binds p53 through MDM2, inhibits the self-ubiquitination of MDM2 E3 ubiquitin ligase, and enhances MDM2-mediated p53 ubiquitination [16]. These results suggest that there may be a common mechanism by which certain ubiquitin ligases, such as UCP and MDM2, differentially catalyze their self-ubiquitination and the ubiquitination of substrates other than themselves.

Viruses often exploit the host ubiquitin system to create a cellular environment for their propagation [37]. HBx binds to DNA damage-binding protein 1 (DDB1) [3]. Cullin-RING ubiquitin ligase 4 (CRL4) uses DDB1 as an adaptor protein [35]. HBx exploits CRL4–DDB1 ubiquitin ligase to target a host antiviral factor for degradation [7]. However, how the E2 enzyme with which CRL4–DDB1 ligase is partnered in vivo has been unclear. Although mammalian Skp1/Cullin-1/F-box and other CRLs can work with either CDC34 or UBC5H family E2 enzymes in vitro, it remains uncertain whether this occurs in vivo [35]. Because the amino terminus (16–50 aa) and the middle domain (85–119 aa) of HBx bind UCP (this study) and DDB1 [3], respectively, UCP is likely partnered with CRL4–DDB1 E3 ligase in cells infected with HBV. It is highly likely that the HBx-bridged CRL4/DDB1–UCP complex targets host restriction factors for degradation. *Cullin*4A amplification has been found in various tumors including HCC [21]. Therefore, our results lead us to speculate on a mechanism by which CRL4 E3 ligases are partnered with E2 enzymes and this may help to identify novel factors associated with tumorigenesis.

The N-terminus (1–50 aa) of HBx alone, but not the transactivation domain (51–154 aa), possesses transforming ability [11]. The N-terminal domain overcomes oncogene-induced senescence by stabilizing full-length HBx [31]. Here, we showed that UCP binds to both the amino termini of HBx (1–50 aa) adr and ayw (Supplementary Fig. 3f) and thereby stabilizes full-length HBx (Fig. 4f, and Supplementary Fig. 5, 6d). Pin isomerase binds HBx in a phosphorylation-dependent manner and stabilizes HBx [32]. Because HBx and UCP, which were expressed in bacteria, interacted with each other (Fig. 3b, d, and Supplementary Fig. 3), phosphorylation of HBx did not appear to be required for the interaction. Thus far, UCP is the only host factor to bind to the amino-terminal regulatory domain of HBx (1–50 aa) serotype- and phosphorylation independently and to stabilize HBx. In HBV-associated HCC, the *HBx* gene is frequently conserved and HBx expression is preferentially maintained [14, 33, 34, 41]. Lifelong and continuous expression of HBx is important for HCC development [20]. UCP is highly expressed in HBx-TG mice and is frequently detected in HBV-positive HCCs (Fig. 1). Thus, our findings suggest that UCP likely enables HBx protein in the liver to be stably maintained at high levels and thereby promotes HBx-mediated HCC development.

In the constitutively HBx-expressing HepG2 and NIH3T3 cell lines, we identified HBx-mediated increases of UCP levels (Fig. 6a, and Supplementary Fig. 6a, d). The HBx-expressing cell lines grew more rapidly than the parental cell line when UCP was additionally expressed (Fig. 6b and Supplementary Fig. 6c). This discrepancy in the growth rate of the cell lines in culture and mice might reflect the nature of actively dividing cells in culture, which can diminish the ability to measure the proliferative effect of HBx [25]. HBx/UCP-mediated stabilization of the HIF-α proteins might also have contributed to the rapid growth of HBx-expressing cells in mice, because HIF-α proteins can induce vigorous angiogenesis in mice and thereby supply adequate nutrients and oxygen necessary for cellular growth. Ad-shUCP decreased the UCP level in the HBx-expressing HepG2 cell line and inhibited the growth of that cell line in cells and mice (Fig. 6c–h). These results suggest that the protein levels of both HBx and UCP are critical for the proliferative effect of HBx on cells, and UCP plays a key role in hepatocarcinogenesis by HBx. In this regard, low levels of HBx and UCP expression may partly account for why some HBx-transgenic mice do not develop liver tumors spontaneously.

HIF-α proteins are stabilized not only in hypoxic condition, but also in non-hypoxic condition [4] [38],]. Indeed, HIF-1α has been shown to be hypoxia-independently overexpressed in preneoplastic hepatocytic lesions from a very early stage during hepatocarcinogenesis in mice and humans [42]. The expression of HIF-1α is significantly higher in HCC with microscopic venous invasion than HCC without microscopic venous invasion [13]. HIF-1α was identified as a biomarker of lymph node metastasis in HCC [48]. HIF-2α expression is positively correlated with invasive HCC features such as tumor size, portal vein invasion, capsule infiltration, necrosis, and intrahepatic metastasis [1, 2]. Both HIF-1α and HIF-2α are involved in inducing the expression of many genes associated with metastasis in HCC [47]. Thus, these previous reports support our findings that the HBx/UCP-mediated stabilization of HIF-α proteins is responsible for the increased invasion and metastasis ability of the HBx-expressing cells (Fig. 7).

While UCP (UBE2S) provides E2 function for the APC E3 ubiquitin ligase, the APC ligase targets UBE2S for ubiquitination and degradation during the G1 phase of the cell cycle [46]. Given that HBx binds to UCP and stabilizes it (Figs. 2, 3, 4), it remains to be determined whether HBx influences the cell cycle by modulating APC E3 ubiquitin ligase activity in mitosis and inhibiting APC-mediated degradation of UCP in G1. Furthermore, since HBx is essential for HBV replication [5], it will be interesting to examine how UCP-mediated stabilization of HBx influences HBV replication.

UCP expression was already detected at early stages of HBV-positive HCC samples (Fig. 1c, HCC stages I/II and I/III), suggesting that UCP might play a role in chronic HBV infection. UCP is highly expressed in numerous human tumors, including HCC, mostly at the mRNA level [44, 45]. Here, we provide experimental evidence that UCP is more frequently detected in HBV-positive HCCs than HBV-negative HCCs at the protein level (Fig. 1a–c). Additionally, we showed that HBx and UCP stabilize each other and UCP is directly involved in HBx-mediated tumor growth and metastasis (Figs. 6, 7).

In conclusion, HBx and UCP interacted and stabilized each other by inhibiting ubiquitination. HBx promoted cellular proliferation and metastasis via UCP. Thus, our findings may help to determine how chronic HBV infection leads to HCC and might be useful for the development of a novel, therapeutic modality to treat HBV-related HCC.

## Materials and methods

### Cell culture and transfection

Human renal carcinoma (RCC) 786-O, human liver cancer SK-hep1, human embryonic kidney 293T, mouse B6F10 melanoma, and mouse embryonic fibroblast NIH3T3 cells were purchased from the American Type Culture Collection. RCC4 and RCC4-pVHL cells^16^ were purchased from the European Collection of Authenticated Cell Cultures. The 786-O-HA-pVHL cell line has been previously described [15]. The human CK-K1, HLK3, and SHJ-1 liver cancer cell lines were supplied by Dr. Dae-Ghon Kim (Chonbuk National University Medical School and Hospital, Jeonju, Republic of Korea). RCC cell lines were maintained in high-glucose Dulbecco’s modified Eagle medium (DMEM, #11965-084, GIBCO BRL, Gaithersburg, MD, USA) with 10% fetal bovine serum (#16000044, GIBCO BRL) and antibiotic–antimycotic (#15240112, GIBCO BRL) in a humidified incubator under 5% CO_2_ at 37 °C. CK-K1, HLK3, SHJ-1, SK-hep1, 293T, B16F10, and NIH3T3 cells were maintained in low-glucose DMEM (#12320-032, GIBCO BRL) with 10% FBS and antibiotics.

To construct cell lines that constitutively express F-HBx, cells were transfected with F-HBx or an empty vector as a control and cultured with 1 mg/ml geneticin (#11811031, GIBCO BRL) for 2 weeks for single-colony selection. DNA transfection was carried out using Lipofectamine (#18324020, Invitrogen, Carlsbad, CA, USA) according to the manufacturer’s protocol.

### Antibodies and reagents

FLAG-tag and β-actin antibodies were purchased from Sigma-Aldrich (St Louis, MO, USA). GST, His-tag, HIF-2α, fibronectin, Slug, Snail and vimentin antibodies were purchased from Santa Cruz Biotechnology (Dallas, TX, USA). HA-tag antibody was purchased form AbFrontier (Seoul, Korea). VHL, HIF-1α, E-cadherin, occluding, and Ki-67 antibodies were purchased from BD Pharmingen (San Jose, CA, USA). HBx antibody was purchased from abcam (Cambridge, MA, USA). The proteasome inhibitor, MG132 (#C2211) and protein synthesis inhibitor, cycloheximide (#01810), were purchased from Sigma-Aldrich. The list of antibodies is described in Supplementary Table 1. The anti-UCP antibody was generated as reported previously [15].

### Plasmids

HBx was amplified by PCR from the pCMV-HBx-3Flag plasmid and cloned into the *Eco*R1/*Xho*1 site of pGEX4T1. HBx mutants were cloned into the *Spe*1/*Not*1 sites of pEBG. For expression of F-HBx in bacteria, HBx was PCR-amplified from pCMV-HBx-3Flag and cloned into the *Nco*1/*Xho*1 site of pET28a. The N-terminal 50 amino acids of HBx serotype ayw or adr were PCR amplified from pCMV-HBx-3Flag (ayw) or pRC-CMV-HBx (adr) plasmids and cloned into the *Eco*R1/*Xho*1 site of pGEX4T1. UCP mutants were cloned into the *Bam*H1/*Not*1 sites of pEBG. The UCP and pVHL expression vectors were constructed as previously described [15]. The sequences of all plasmids constructed in this study were verified by direct sequencing before use (data not shown). Adenoviral vectors have been previously described [15].

### Measurement of cell viability

DNA synthesis was assessed by measuring the incorporation of 5-bromo-2-deoxyuridine (BrdU) into cellular DNA using a BrdU cell proliferation assay kit (#6813, Cell signaling, Danvers, MA, USA) according to the manufacturer’s protocol. Cells were transduced with Ad-shUCP or Ad-shControl with a MOI of 200, cultured for 24 h, and seeded at 3 × 10^3^/well in 96-well plates. Proliferation of cells was analyzed three times independently after 12, 48, and 72 h.

For the cell viability assay, cells were seeded at 3 × 10^3^/well in 96-well plates, cultured for 24 h, and incubated in fresh culture medium containing 10 μl of CytoX (Roche, Basel, Switzerland) for 1 h at 37 °C. Cell viability was determined by measuring the optical density (OD) at 450 nm using a microplate reader.

### Cytofluorimetric analysis of apoptosis and autophagy using the Muse™ cell analyzer

For confirming the cell cycle effect of HBx and UCP expression, the cells were transduced with Ad-F-UCP or Ad-LacZ at a 50 MOI for 24 h. Then, the cells were fixed with 70% ethanol, washed with ice-cold PBS, and the cells were incubated with a Muse cell cycle kit (#MCH100106, EMD Millipore, Hayward, CA, USA) at RT for 30 min and analyzed on a Muse™ cell analyzer (EMD Millipore). Data were quantified using the Muse™ analysis software (EMD Millipore).

### Reverse transcription-PCR and quantification RT-PCR

Total RNA was extracted from cells using an easy-spin RNA extraction kit (#17221, Intron, Seoul, Korea). cDNA was synthesized using 3 μg of total RNA, reverse transcriptase (#2680A, TakaRa, Japan), and oligo(dT) primer. The mRNA expression of target genes was analyzed by RT-PCR and qRT-PCR (AriaMx Real Time PCR System, Agilent Technologies) using specific primers indicated in Supplementary Table 2 and 3.

### Western blotting (WB)

Cells were lysed on ice in RIPA buffer (50 mM Tris–HCl, pH 7.5, 150 mM NaCl, 0.5 mM EDTA, 1% NP40, 0.1% SDS, and 1 mM PMSF). Proteins were separated by SDS-PAGE and transferred to a polyvinylidene fluoride membrane (Millipore), which was incubated in blocking buffer [5% skim milk in PBS, 0.1% Tween-20 (PBST)] for 1 h at room temperature. The membrane was incubated with specific primary antibodies in PBST for 2 h at room temperature or overnight at 4 °C. Subsequently, the membrane was incubated with the secondary antibody in PBST containing 0.5% skim milk for 1 h at room temperature. Proteins were visualized using a chemiluminescence kit (# 16021, Intron).

### Purification of recombinant proteins

pGEX4T1 vector-based GST-fusion proteins or pET28a vector-based His-fusion proteins were expressed and purified following the manufacturer’s instruction (# 30210, Qiagen, Hilden, Germany). Bacterial cells were cultured to OD_600_ 0.5 and then induced with 1 mM IPTG for 2 h at 37 °C. The cells were harvested by centrifugation at 8000 rpm for 5 min at 4 °C. The cells expressing GST- or His-fusion proteins were re-suspended in lysis buffer (PBS, 1% Triton X-100, protease inhibiter cocktail, and 1 mM PMSF or 20 mM Tris–HCl, pH 7.9, 500 mM NaCl, 5 mM imidazole) and sonicated on ice. Cell lysates were centrifuged at 13,000 rpm for 10 min at 4 °C. The supernatants containing GST- or His-fusion proteins were incubated with Glutathione Sepharose 4B (#17075601, GE Healthcare, Little Chalfont, UK) or Ni–NTA agarose beads for 2 h at 4 °C. Bead-bound GST-fusion proteins were washed with PBS and eluted with a buffer (50 mM Tris, pH 8.8, 1 mM EDTA, 25 mM reduced glutathione, and 1 mM PMSF). Bead-bound His-fusion proteins were washed with wash buffer (20 mM Tris, pH 7.9, 500 mM NaCl, and 60 mM imidazole) and eluted with a buffer (20 mM Tris, pH 7.9, 250 mM NaCl, and 500 mM imidazole). After dialysis, the purified proteins were stored at − 70 °C.

### Human samples

Primary HCC and normal liver tissues were obtained from patients who underwent surgery for HCC at Chonbuk National University Hospital (Jeonju, Korea). Informed consent was obtained from patients for the use of specimens for research purposes only. Specimens were frozen in liquid nitrogen and stored until use.

### Mouse samples

Homozygote-transgenic mice carrying the HBx adr gene (C57BL/6xDBA) [50] were maintained in a laboratory animal facility at the Korea Research Institute of Bioscience and Biotechnology (KRIBB, Daejeon, Korea) under specific pathogen-free conditions. HBx-TG mice at various ages and age-matched wild-type controls were maintained in a barrier room. Food and water were supplied ad libitum. Mice were maintained in accordance with the guidelines for Animal Care and Use, KRIBB.

### Immunohistochemistry (IHC) assay

IHC was performed on paraffin human HCC tissues microarrays (containing 16 pairs of normal liver and 32 pairs of HCC tissues from cat. LV20812a, US Biomax Inc., MD, USA) and mouse tumor xenografts. IHC staining was performed using Vectastain Elite ABC-HRP kit (#PK-7200, Vector lab., CA, USA) and DAB substrate kit (#SK-4100, Vector lab.) following the manufacturer’s instructions. The image was pictured by Olympus microscopy under a 10 × or 20 × magnification and quantified using Image J software.

### Pull-down assay

Cells were lysed in NET gel buffer (50 mM Tris–HCl, pH 7.5, 150 mM NaCl, 0.1% NP-40, 1 mM EDTA, pH 8.0) supplemented with complete protease inhibitor cocktail (Roche, # 4693159001). The cell lysates were centrifuged at 13,000 rpm for 10 min at 4 °C. The supernatants were incubated with Glutathione Sepharose or Ni–NTA agarose beads for 2 h at 4 °C with gentle shaking. After incubation, the beads were washed three times with 1 ml of PBST buffer. Bead-bound proteins were analyzed by WB.

### Immunoprecipitation (IP) assay

Cell lysates or reaction mixtures were immuno-precipitated with appropriate antibodies or a FLAG-specific agarose gel for 2 h at 4 °C. For HA-tagged fusion protein, cell lysates were immune-precipitated with 2 μg of HA antibody, incubated at 4 °C overnight, and then incubated with 20 μl of Protein-A Sepharose bead (10% slurry in PBS) at 4 °C for 2 h. The precipitates or bead-bound proteins were washed and analyzed by WB.

### In vitro and in vivo ubiquitination assays

The reaction mixture for the self-ubiquitination assay (50 μl) contained 0.3 μg of GST-UCP or His-UCP, 0.5 μg of E1 (Sigma-Aldrich, #SRP6147), and 1.25 μg of His-Ub in a reaction buffer (25 mM Tris–Cl, pH 7.5, 1 mM ATP, 5 mM creatine phosphate, 0.5 μg/ml creatine phosphate kinase, 1 mM DTT, 5 mM MgCl_2_, and 0.5 μg/ml ubiquitin aldehyde). The reaction mixture for the pVHL ubiquitination assay (50 μl) contained the indicated amount of His-UCP, 5 μg of GST-pVHL or GST, 1.25 μg of F-Ub, and 0.5 μg of E1. The reaction mixtures were incubated at 37 °C for 1 h and analyzed.

An in vivo ubiquitination assay was performed as described previously [15]. Briefly, cells were treated with MG132, lysed in a buffer (50 mM Tris–HCl, pH 7.5, 150 mM NaCl, 0.5 mM EDTA, 0.1% NP40, and 1 mM PMSF), and sonicated. The supernatants were obtained by centrifugation of cell lysates at 13,000 rpm for 30 min at 4 °C. A pull-down assay was performed with the supernatants as described above.

### Cell invasion assay

An invasion assay was performed as previously described [23]. Invaded cells were stained with H&E and observed under a microscope.

### In vivo xenograft assay

Five-week-old female BALB/c nude mice were purchased from SLC Japan (Hamamatsu, Japan) and maintained in accordance with the guidelines of the Institutional Review Committee for Animal Care and Use, KRIBB. Cells were injected into nude mice at the numbers described in the figure legends. Tumor size was measured as reported previously (Jung et al. [17]).

### In vivo metastasis assay

For the orthotopic metastasis assay, nude mice were anesthetized with 2,2,2-tribromoethanol (Avertin; # T48402, Sigma-Aldrich), and 1 × 10^6^ cells suspended in 50 µL of Matrigel (#354230, BD Biosciences, San Jose, CA, USA) were directly injected into the liver. The area around the injection site was cleaned with saline buffer to avoid seeding of refluxed tumor cells into the abdominal cavity. At 4 weeks after the injection, tumors that had metastasized to organs were analyzed in the mice.

For the experimental metastasis assay, the cells in 100 µl of PBS were injected into the tail vein of 5-week-old female BALb/c nude mice. The mice were killed after 2 weeks and tumors metastasized to the lungs were analyzed.

### TCGA RNA-seq expression and clinical data

Liver Hepatocellular Carcinoma (TCGA-LIHC) RNA-seqv2 data (HTSeq-FPKM) and clinical datasheet were obtained from the TCGA data portal (Jun 28, 2017, Jan 30, 2018, respectively). Data were preprocessed with TCGAbiolinks package (Colaprico et al. [6]) in R, and biomaRt package [9] was used to convert the ensembl to a symbol. Primary Solid Tumor 371 samples from 49 patients contained paired normal liver samples were included. The differential expression gene (DEG) analysis was performed on two types of samples: 81 patients infected with hepatitis B virus, and 227 patients without hepatitis B virus defined as control data. The log2 transformation of FPKM was adopted in all plots.

### Statistical analysis

Student’s *t* test was used to analyze the association between hepatitis B virus infection and Ube2 s expression. For survival analysis, we used the Kaplan–Meier method to analyze the correlation between overall survival time and UBE2S expression. The statistical significances of overall survival were determined using the log-rank test. Survival analysis was performed in R (version 3.3.1). Statistical significance was based on a *p* value less than 0.05. The median was used as a cutoff value for classification of patients into high and low expression groups.

### Electronic supplementary material

Below is the link to the electronic supplementary material.

Supplementary material 1 (DOCX 1342 kb)

Supplementary material 2 (DOCX 15 kb)

## Abbreviations

HBV: Hepatitis B virus
HBx: X protein
UCP: Ubiquitin carrier protein
pVHL: von Hippel–Lindau protein
TG: Transgenic
HIF: Hypoxia-inducible factor
RCC: Human renal carcinoma
DMEM: Dulbecco’s modified Eagle medium
FBS: Fetal bovine serum
AA: Amino acid
CDC34: Cell division cycle 34
EMT: Epithelial–mesenchymal transition
H&E: Hematoxylin and eosin
RT-PCR: Reverse transcription polymerase chain reaction
WB: Western blotting
IP: Immunoprecipitation
RCC: Renal carcinoma
PBS: Phosphate-buffered saline
PBST: PBS + 0.1% Tween-20
IHC: Immunohistochemistry
Pin 1: Peptidyl prolyl isomerase
DDB1: DNA damage-binding protein 1
CRL4: Cullin-RING ubiquitin ligase 4
