# Supplementary material for: Stabilization of E2-EPF UCP protein is implicated in hepatitis B virus-associated hepatocellular carcinoma progression
Source: Cell Mol Life Sci. 2019 Mar 22;76(13):2647–62. doi: 10.1007/s00018-019-03066-9 (PMC6586911; doi:10.1007/s00018-019-03066-9)
Supplement: Supplementary file 1 — Supplementary material 1 (DOCX 1342 kb) [file 18_2019_3066_MOESM1_ESM.docx]

**Stabilization of E2-EPF UCP protein is implicated in hepatitis B virus associated hepatocellular carcinoma progression**

**Running title: HBx protein increase the stability of E2-EPF UCP**

Jung Hwa Lim^1^, Dae-Ghon Kim^3^, Dae-Yeul Yu^1^, Hyun Mi Kang^1^,

Kyung Hee Noh^1^, Dae-Soo Kim^1^, Dongmin Park^1^, Tae Kyung Chang^1^,

Dong-Soo Im^1*^ and Cho-Rok Jung^1,2,*^

^1^ Korea Research Institute of Bioscience and Biotechnology (KRIBB),*Daejeon, Republic of Korea*

^2^*University of Science and Technology, Daejeon, Republic of Korea*

^3^*Research Institute of Clinical Medicine, Chonbuk National University Medical School and Hospital, Jeonju, Republic of Korea*

***Corresponding authors:**

Cho-Rok Jung, *Gene Therapy Research Unit, KRIBB, Daejeon, Republic of Korea,*

*tel: +82-42-860-4171, fax: +82-42-860-4597,* E-mail: [crjung@kribb.re.kr](mailto:crjung@kribb.re.kr).

Dong-Soo Im, *Gene Therapy Research Unit, KRIBB, Daejeon, Republic of Korea,*

*tel: +82-42-860-4172, fax: +82-42-860-4597,* E-mail: [imdongsu@kribb.re.kr](mailto:imdongsu@kribb.re.kr)

**Supplementary Figure legends**

**Supplementary Fig. 1. HBx increases UCP level, decreases pVHL level, and regulates the protein level of HIF-2α through pVHL in cells.**

**(a,b)** We transfected 786-0 and 786-0-HA-pVHL cells with F-HBx expression vector (0, 5, 15 μg, -, +, ++), incubated them for 24 h, and prepared whole cell lysates or total-RNAs. We analyzed the lysates by WB as indicated (a), and analyzed the total RNAs by RT-PCR as indicated (b). Protein bands or RT-PCR products were quantified by densitometry and the results are expressed relative to the band intensity of the first or fourth (pVHL panel) lane in Supplementary Fig. 1a or 1b, which was arbitrarily defined as 1. S: short exposure, L: long exposure. Representative data of at least three independent experiments are shown.

**Supplementary Fig. 2. The mRNA level of UCP, VHL, and HIF-1α by HBx expression is no effect in diverse cancer cell lines.**

**(a)** We transfected the indicated cells with FLAG-tagged HBx (F-HBx) expression vector (0, 5 μg, -, +), incubated them for 24 h, and analyzed them by RT-PCR as indicated. **(b)** We transfected the indicated cells with F-HBx expression vector (0, 5, 15 μg, -, +, ++), prepared total RNAs, and analyzed them by RT-PCR as indicated. The mRNA levels are expressed relative to the band intensity of the first lane in supplementary Fig. 2b, which was arbitrarily defined as 1.

**Supplementary Fig. 3. The amino-terminal domain (1-50 aa) of HBx binds to UCP serotype-independently.**

**(a)** Schematic illustration of the HBx protein (1-154 aa) comprising the regulatory (1-50 aa) and transcription factor binding domains (51-154 aa). Below are shown the aa sequences of the regulatory amino-terminus domain from HBV serotypes ayw or adr. Asterisks (*) indicate amino acid sequence difference. aa, Amino acid. **(b)** We transfected 293T cells with F-UCP (10 μg) and each GST-HBx illustrated in Fig. 4e or GST expression vector (10 μg each), incubated them for 24 h, and performed pull-down assays as indicated. **(c)** We transfected 293T cells with F-HBx adr (10 μg) and each GST-UCP illustrated in Fig. 4e or GST expression vector (10 μg each), incubated them for 24 h, and performed pull-down assays as indicated. **(d)** We transfected 293T cells with HA-pVHL (10 μg) and each GST-UCP illustrated in Fig. 4e or GST expression vector (10 μg each), incubated them for 24 h, and performed pull-down assays as indicated. **(e)** Schematic illustration of various GST-HBx adr or GST-UCP expression vectors. The amino-terminus domain (1-50 aa) of HBx is shown in black. The results from the binding assay in the Fig. 4b-d are summarized on the right. **(f)** We mixed His-UCP (1 μg), GST-HBx (1-50 aa) of HBV ayw or adr (2 μg each), or GST proteins (2 μg), incubated them at 4°C for 1 h, and performed pull-down assays as indicated. We repeated the experiments of Fig. 4b-d three times and that of Fig. 4f twice; representative data are shown.

**Supplementary Fig. 4. HBx inhibits ubiquitination of UCP in vitro.**

We performed an in vitro ubiquitination assay with F-HBx (0, 0.5, 3 μg, -, +, ++), His-UCP (0.3 μg), GST-UCPm (3 μg), or GST (3 μg) proteins, and immunoblotted the reaction mixtures as indicated. We used a catalytically inactive GST-UCPm as a substrate, in which cysteine-95 is mutated to alanine. Representative data of at least three independent experiments are shown.

**Supplementary Fig. 5. UCP prolongs HBx half-life.**

**(a)** HLK3 cells stably expressing F-HBx (HLK3-F-HBx) were transfected with GST or GST-UCP expression vector (10 μg each), treated with 100 μg/ml CHX, harvested at the indicated times, and immunoblotted as indicated. We repeated this experiment three times and representative data are shown. **(b)** HBx bands in Supplementary Fig. 5a were quantified by densitometry and are expressed relative to the band intensity of ‘time 0’, which was arbitrarily defined as 100. Data are mean ± S.D.

**Supplementary Fig. 6.** **HBx and UCP cooperatively promote tumor growth in mice.**

**(a,b)** We transfected NIH3T3 cells with F-HBx expression vector (10 μg), and independently selected the indicated cell lines in the presence of culture medium including geneticin (1 mg/ml). We prepared whole cell lysates or total RNAs from parental or HBx-expressing NIH3T3 cell lines and immunoblotted them as indicated (a) or performed an RT-PCR assay using total RNAs as indicated (b). RT-PCR products were quantified by densitometry and the results are expressed relative to the band intensity of the first lane in Supplementary Fig. 6b, which was arbitrarily defined as 1. Representative data of at least two independent experiments are shown. **(c)** We transduced or not parental or F-HBx-expressing NIH3T3 cells with Ad-F-UCP or Ad-LacZ at the indicated MOI, incubated them for 24 h, and treated them with WST1 for 1 h. Cell viability was then determined. We repeated this assay twice, each in triplicate. Data are mean ± S.D. **(d)** We transduced or not parental or F-HBx-expressing NIH3T3 cells with Ad-F-UCP or Ad-LacZ at the indicated MOI, incubated them for 24 h, and analyzed them by WB as indicated. Representative data of at least three independent experiments are shown. **(e, f)** We transduced parental or F-HBx-expressing NIH3T3 cells (0.5 × 10^7^) with Ad-F-UCP or Ad-LacZ at a MOI of 50, and incubated them for 24 h, and injected them into nude mice (N=5 per group) subcutaneously. Tumor volume was measured every three days and tumors were excised from mice at 36 days after inoculation and photographed. Tumors were not formed in some mice. We repeated this xenograft assay three times and representative data are shown. **(g)** We transduced parental or F-HBx-expressing NIH3T3 with Ad-shUCP or Ad-shControl (ref. 12) at a MOI of 200, incubated them for 24 h, and analyzed them by WB. **(h, i)** We transduced or not parental or F-HBx-expressing NIH3T3 cells with Ad-shUCP or Ad-shControl at a MOI of 200, incubated them for 24 h, and injected them (1.5 × 10^7^ cells/mouse) into nude mice (N = 5 per group) subcutaneously. Mice were sacrificed at day 38, and tumors were then excised from mice and photographed (h). Tumor growth was monitored by measuring tumor volume with digital caliper at the indicated times (i). We repeated the assay of Fig. 6h,i twice; representative data are shown.

**Supplementary Fig. 7. HBx and UCP cooperatively promote metastasis in an orthotopic metastasis mouse model.**

**(a,b)** We established SHJ-1 hepatoma cells stably expressing F-HBx (SHJ-1-F-HBx), transduced them with Ad-F-UCP or Ad-LacZ at a MOI of 25, and incubated them for 24 h. We mixed the resulting SHJ-1-F-HBx cells with Matrigel, and directly injected 50 μl of the mixture including 1 × 10^6^ SHJ-1-F-HBx cells into the mouse liver as indicated (a). After four weeks, the liver was excised from mice with or without tumor cell injection, fixed, sectioned and stained with H&E to confirm that the injected tumor cells were stably grafted (b). Scale bar, 100 μm. **(c-e)** We injected 50 μl of the Matrigel mixture including 1 × 10^6^ SHJ-1-F-HBx cells transduced with or without Ad-F-UCP or Ad-LacZ at a MOI of 25 into the liver of the mice as described in Supplementary Fig. 8a,b. At four weeks after tumor cell injection, we weighed the mice, excised the liver and lung, and weighed (c) and photographed (d) them. The total number of tumors that metastasized from the mouse liver to other organs or tissues were counted (e). We repeated these experiments twice, and representative data are shown.

**Supplementary Fig. 8. HBx and UCP cooperatively promote cell invasion in vitro assay.**

**(a,b)** We transduced or not parental or F-HBx expressing NIH3T3 cells with phosphate-buffered saline (PBS), Ad-shUCP or Ad-shControl at a MOI of 200, incubated them for 48 h, and performed an invasion assay with each group of the cells in triplicate. Invaded cells were stained with H&E, and photographed under a microscope (scale bar 100 μm) (a). Representative images are shown. Invaded cells are counted in five random fields and graphed (b). Data are mean ± S.D. (i.e. **p* < 0.05, ***p* < 0.01)

**
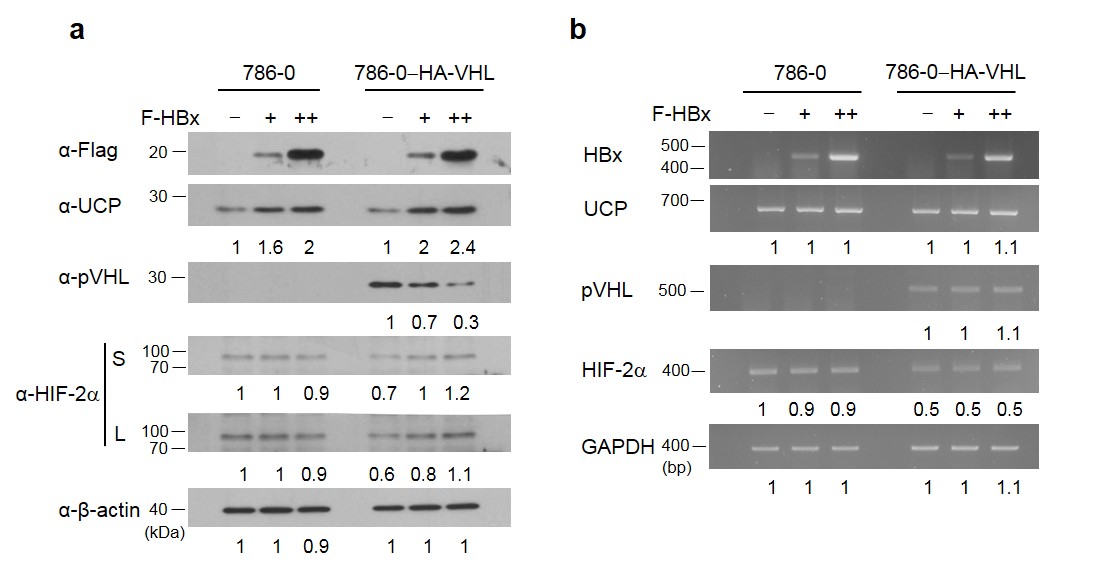
**

**Supplementary Figure 1**


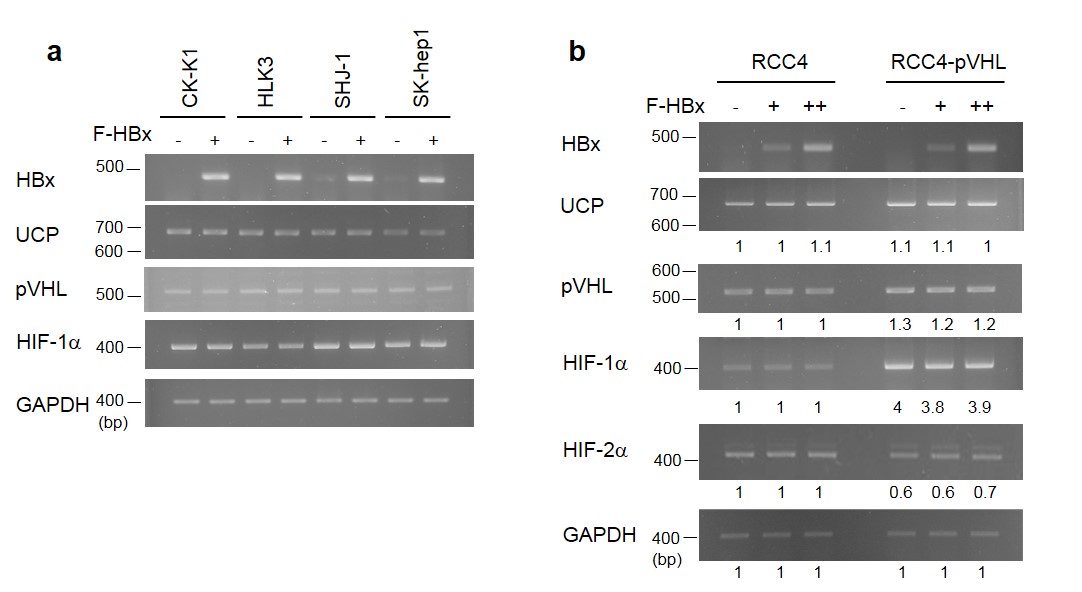


**Supplementary Figure 2**

**
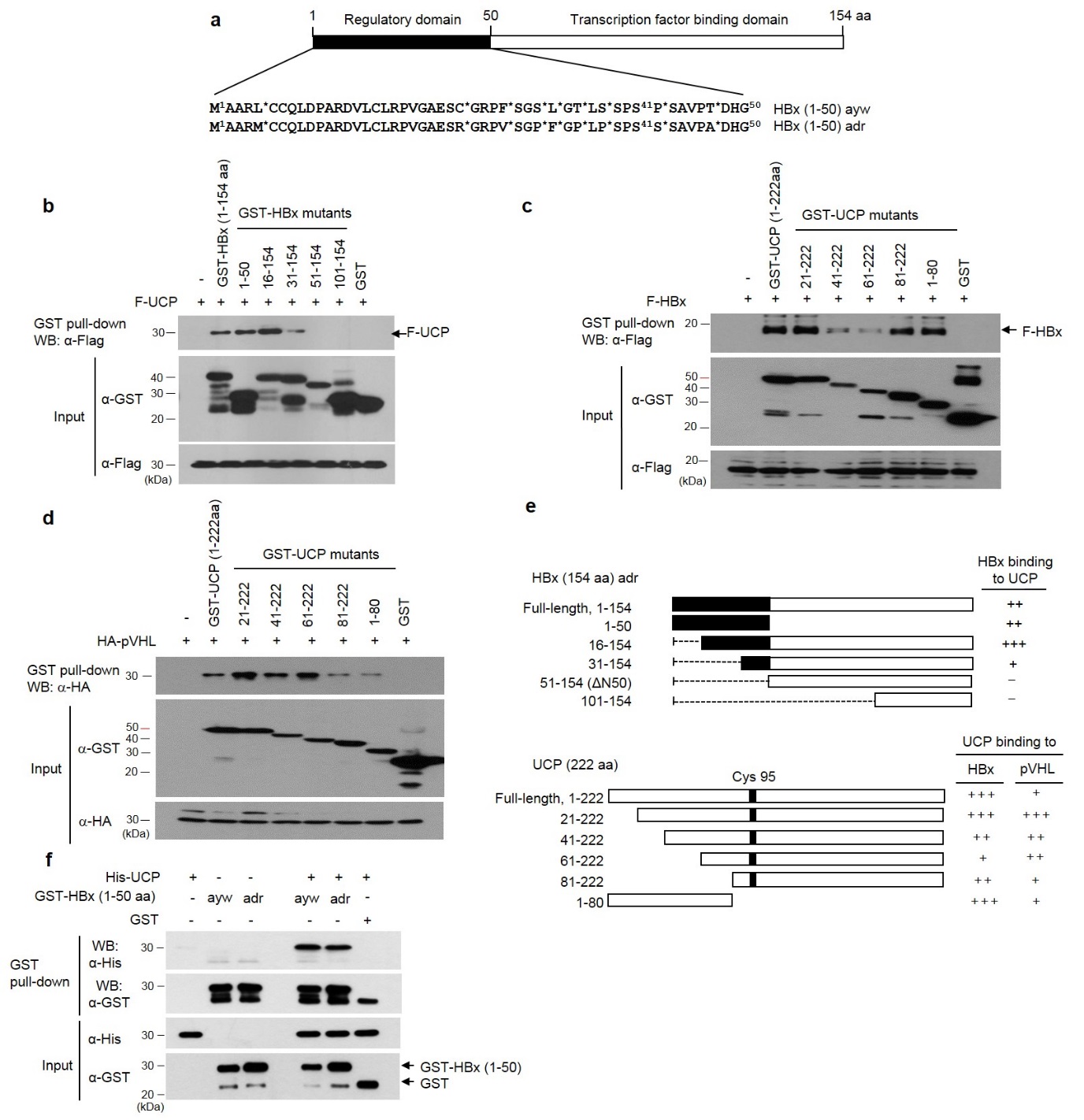
**

**Supplementary Figure 3**

**
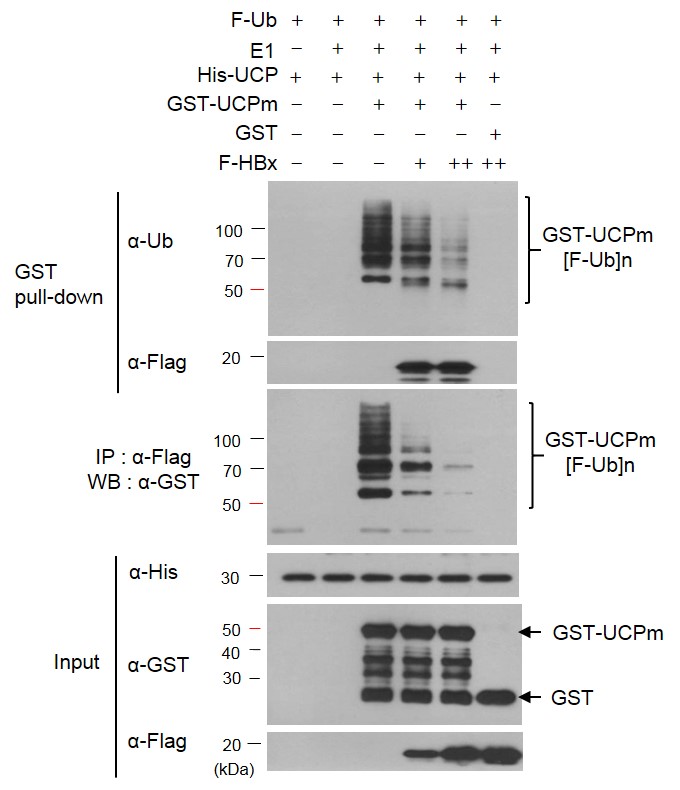
**

**Supplementary Figure 4**


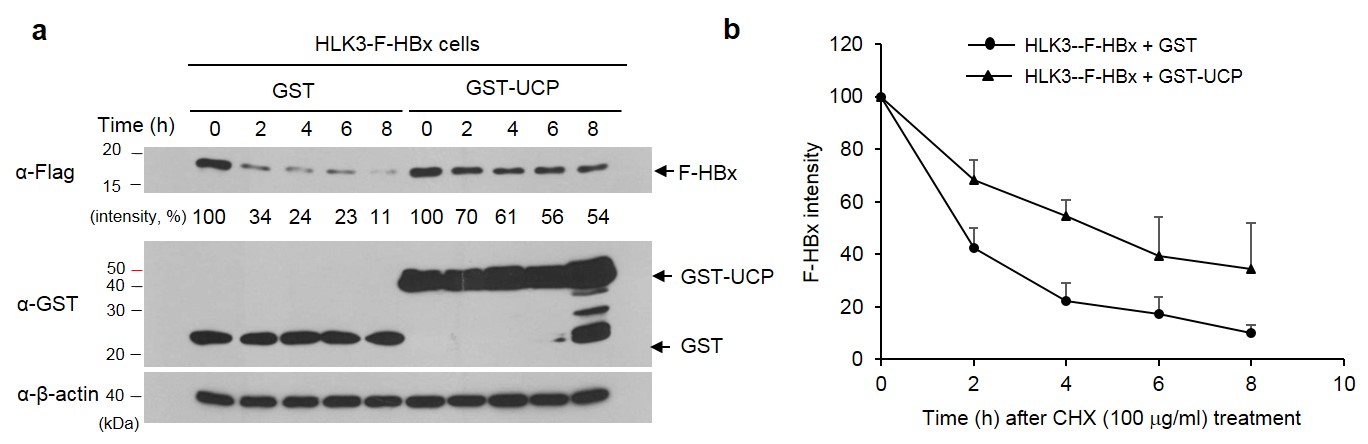


**Supplementary Figure 5**


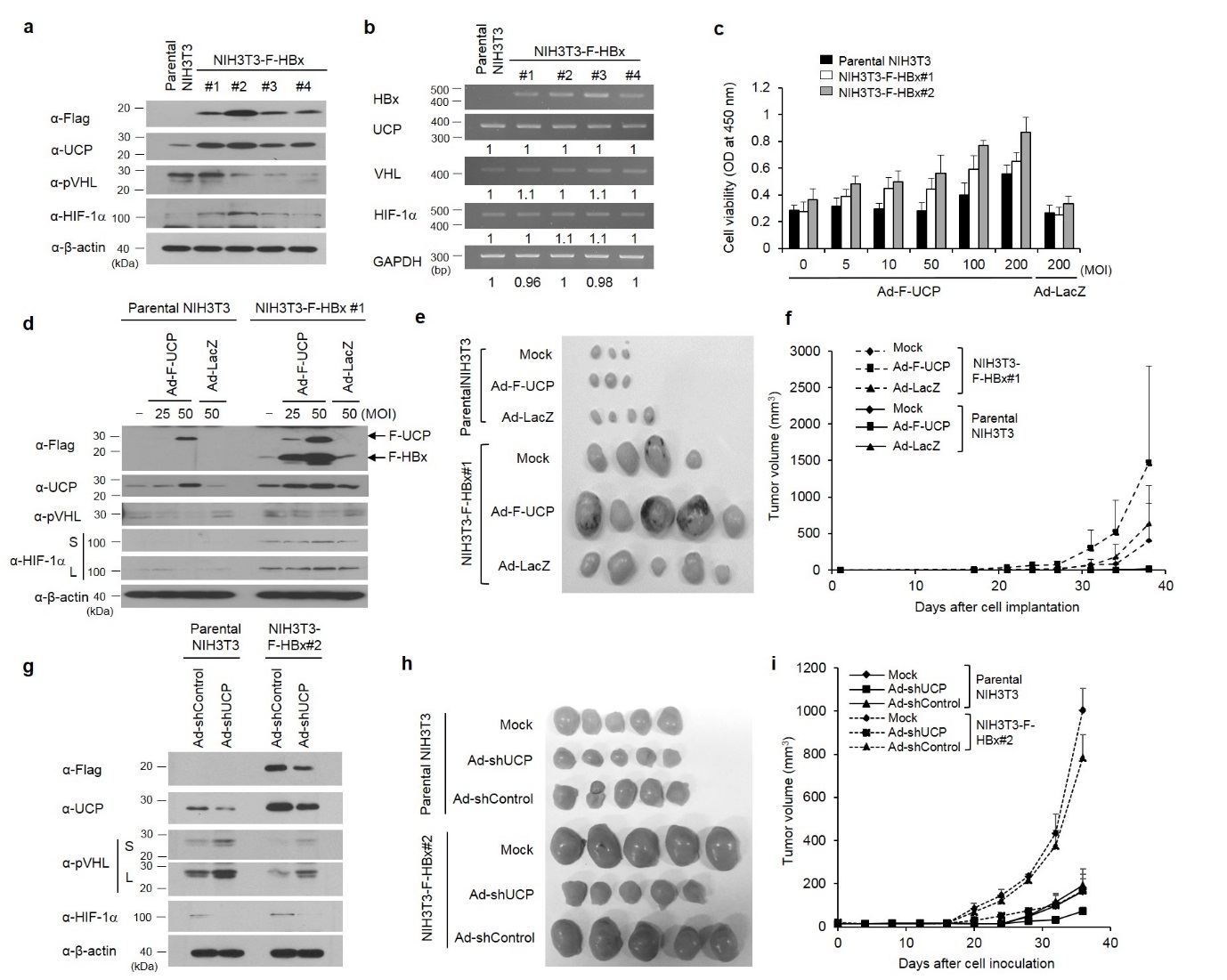


**Supplementary Figure 6**

**
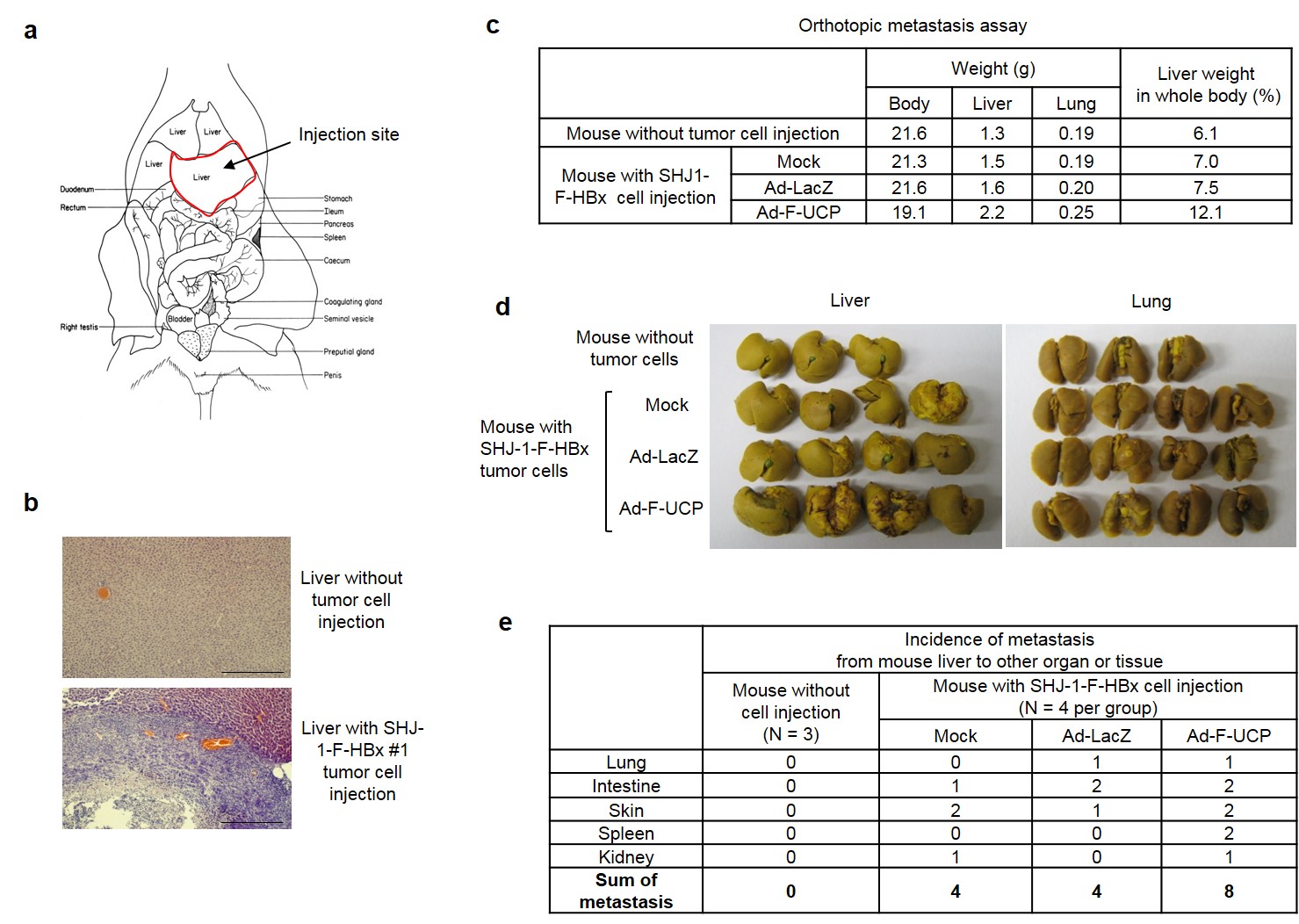
**

**Supplementary Figure 7**

**
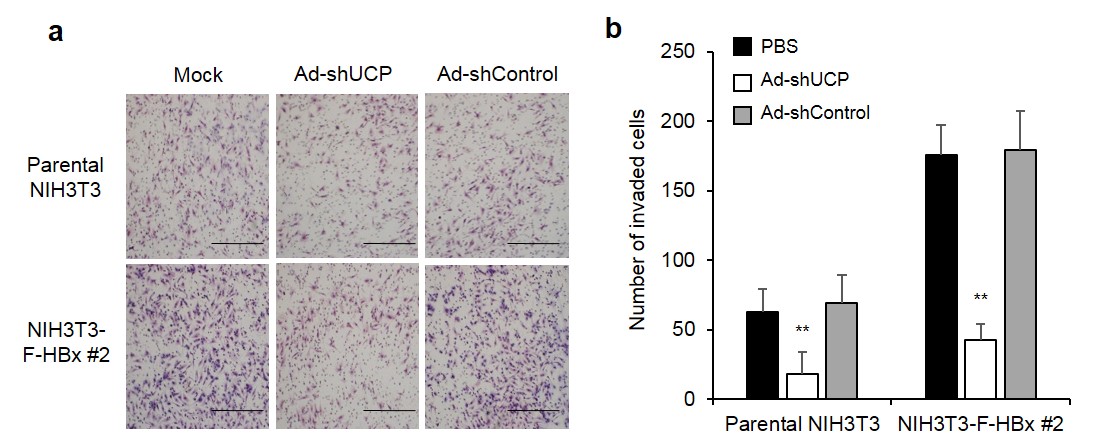
**

**Supplementary Figure 8**
