# Supplementary material for: Stabilization of E2-EPF UCP protein is implicated in hepatitis B virus-associated hepatocellular carcinoma progression
Source: Cell Mol Life Sci. 2019 Mar 22;76(13):2647–62. doi: 10.1007/s00018-019-03066-9 (PMC6586911; doi:10.1007/s00018-019-03066-9)
Supplement: Supplementary file 2 — Supplementary material 2 (DOCX 15 kb) [file 18_2019_3066_MOESM2_ESM.docx]

**Supplementary Table S1. Antibodies sources**

| **Genes** | **Source** | **Host** | **Cat #** |
| --- | --- | --- | --- |
| Flag | Sigma-Aldrich | Mouse | F1804 |
| GST | Santa-cruz | Mouse | sc-138 |
| His probe | Santa-cruz | Mouse | sc-8036 |
| HA probe | AbFrontier | Mouse | LF-MA0048 |
| VHL | BD Pharmingen | Mouse | 564183 |
| HIF-1α | BD Pharmingen | Mouse | 610958 |
| HIF-2α | Santa Cruz | Mouse | sc-28706 |
| Fibronectin | Santa Cruz | Mouse | sc-8422 |
| Slug | Santa Cruz | Mouse | sc-166476 |
| SNAI-1 | Santa Cruz | Rabbit | sc-28199 |
| Vimentin | Santa Cruz | Mouse | sc-6260 |
| E-cadherin | BD Pharmingen | Mouse | 610404 |
| occludin | BD Pharmingen | Mouse | 611090 |
| Ki-67 | BD Pharmingen | Mouse | 556003 |
| HBx  (for IHC on mouse xenografts) | Santa Cruz | Mouse | sc-57760 |
| HBx  (for IHC on human HCC tissues array) | Abcam | Rabbit | ab39716 |
| β-actin | Sigma-Aldrich | Mouse | A5441 |

**Supplementary Table S2. Primer sequences and conditions for conventional RT-PCR**

| **Genes** | **Forward Primer (5’-3’)** | **Reverse Primer (5’-3’)** |
| --- | --- | --- |
| HBx | ATGGCTGCTAGGCTGTGC | TTAGGCAGAGGTGAAAAA |
| UCP  (human) | ATGAACTCCAACGTGGAGAA | CTACAGCCGCCGCAGCGC |
| pVHL  (human) | ATGCCCCGGAGGGCGGAG | TCAATCTCCCATCCGTTG |
| HIF-1α  (human) | AGAAAAAGATAAGTTCTGAACGTCG | ATTTCCTCATGGTCACATGGATG |
| HIF-2α  (human) | AGCAAAGACATGTCCACAGA | TCTTGGTCATGTTCTCGGAG |
| GAPDH  (human) | ATGGGGAAGGTGAAGGTCGG | TGGTTCACACCCATGACGAA |
| UCP  (mouse) | ACCCACCTGATGGCATTAAA | TGGATTTCTGTGAGCAGACG |
| pVHL  (mouse) | CTCAGGTCATCTTCTGCAACC | TCCTCTTCCAGGTGCTGACT |
| HIF-1α  (mouse) | AAAAACAGAGACGAAGGACA | TGCTAAATCGGAGGGTATTA |
| GAPDH  (mouse) | ATGACAACTTTGGCATTGTG | GAAGAGTGGGAGTTGCTGTT |

**Supplementary Table S3. Primer sequences and conditions for conventional qRT-PCR**

| **Genes**  **(Human)** | **Forward Primer (5’-3’)** | **Reverse Primer (5’-3’)** |
| --- | --- | --- |
| *HBx* | GTCGCTTGGGACTTTCTCGT | GGAGTCCGCGTAAAGAGAGG |
| *UCP* | TCAAGTGCCTGCTGATCCAC | TGGATCTCTGTGAGCAGACG |
| *VHL* | GATGCAGGGACACACGATGG | ACTAGGCTCCGGACAACCTG |
| *HIF1a* | GACAAGCCACCTGAGGAGAG | CACGCGGAGAAGAGAAGGAA |
| *TUBB* | GTGGTACGGAAGGAGGTCGAT | GGCGGAACATGGCAGTGAAC |
